# Supplementary figures and images for: Hepatic metabolite responses to 4-day complete fasting and subsequent refeeding in rats (part 2 of 2)
Source: PeerJ. 2022 Sep 20;10:e14009. doi: 10.7717/peerj.14009 (PMC9504452; doi:10.7717/peerj.14009)

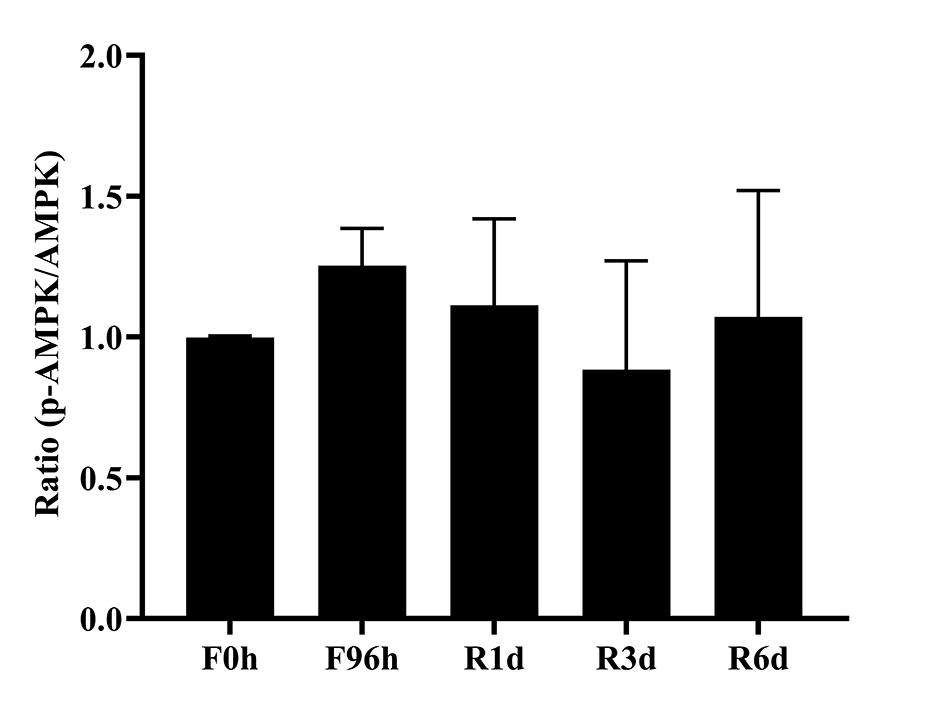

Supplement: Supplemental Information 1 [file peerj-10-14009-s001.zip › Raw data/Figure 7I.png]

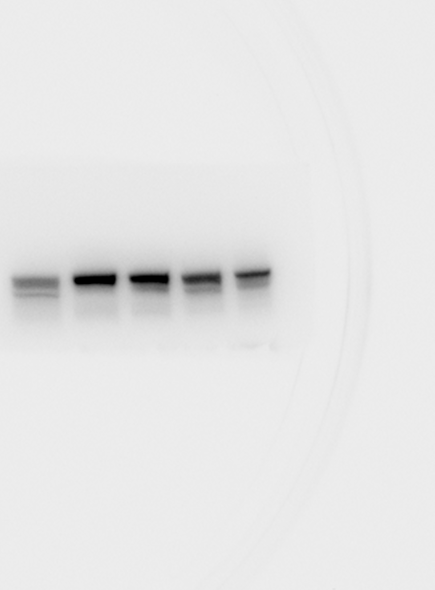

Supplement: Supplemental Information 1 [file peerj-10-14009-s001.zip › Raw data/Figure 7J mTOR-1.png]

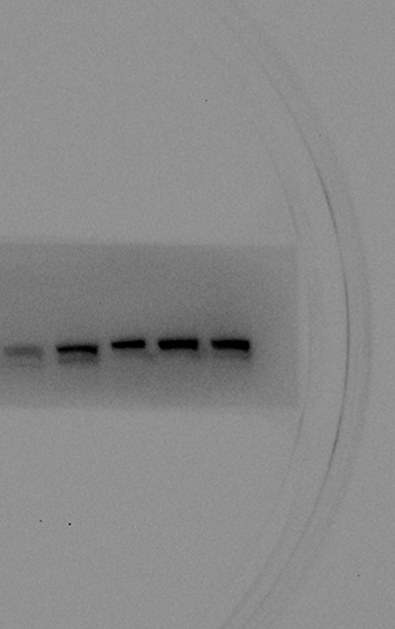

Supplement: Supplemental Information 1 [file peerj-10-14009-s001.zip › Raw data/Figure 7J mTOR-2.png]

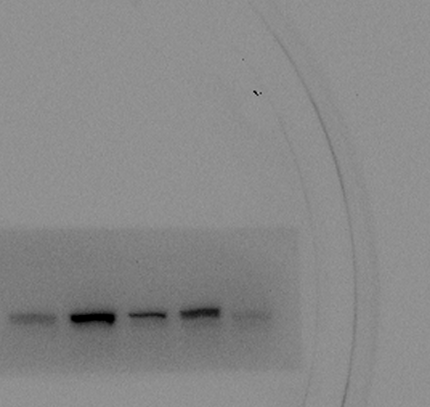

Supplement: Supplemental Information 1 [file peerj-10-14009-s001.zip › Raw data/Figure 7J mTOR-3.png]

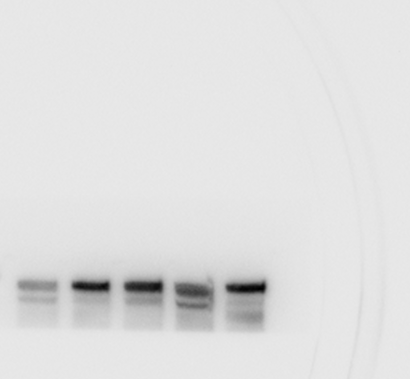

Supplement: Supplemental Information 1 [file peerj-10-14009-s001.zip › Raw data/Figure 7J mTOR-4.png]

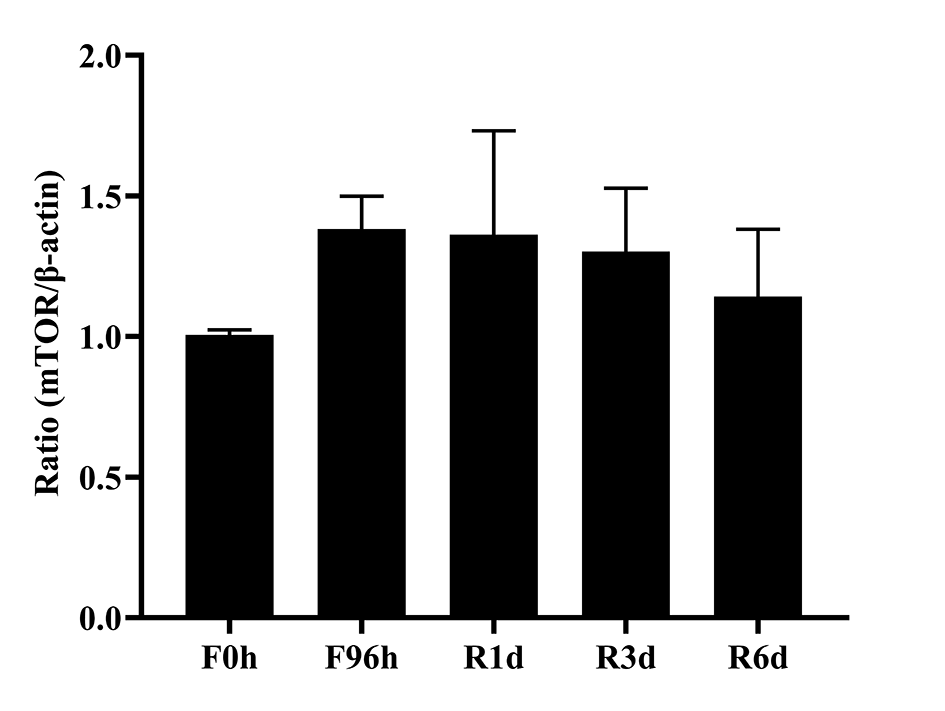

Supplement: Supplemental Information 1 [file peerj-10-14009-s001.zip › Raw data/Figure 7J.png]

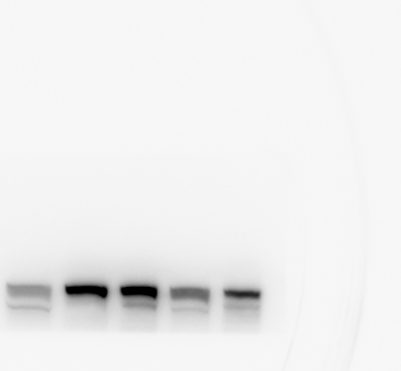

Supplement: Supplemental Information 1 [file peerj-10-14009-s001.zip › Raw data/Figure 7K p-mTOR-1.png]

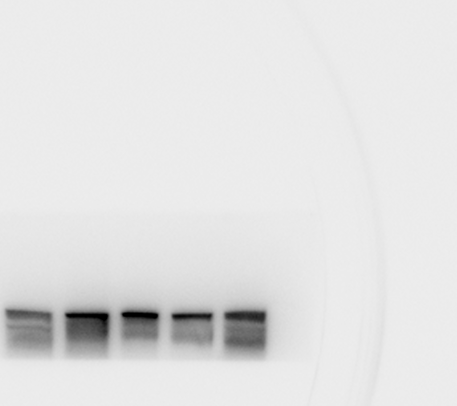

Supplement: Supplemental Information 1 [file peerj-10-14009-s001.zip › Raw data/Figure 7K p-mTOR-2.png]

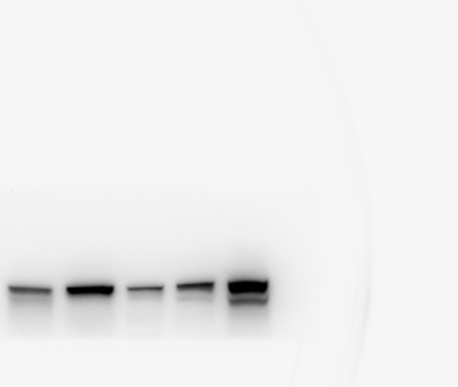

Supplement: Supplemental Information 1 [file peerj-10-14009-s001.zip › Raw data/Figure 7K p-mTOR-3.png]

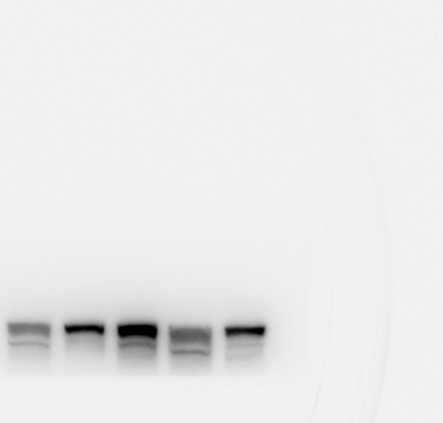

Supplement: Supplemental Information 1 [file peerj-10-14009-s001.zip › Raw data/Figure 7K p-mTOR-4.png]

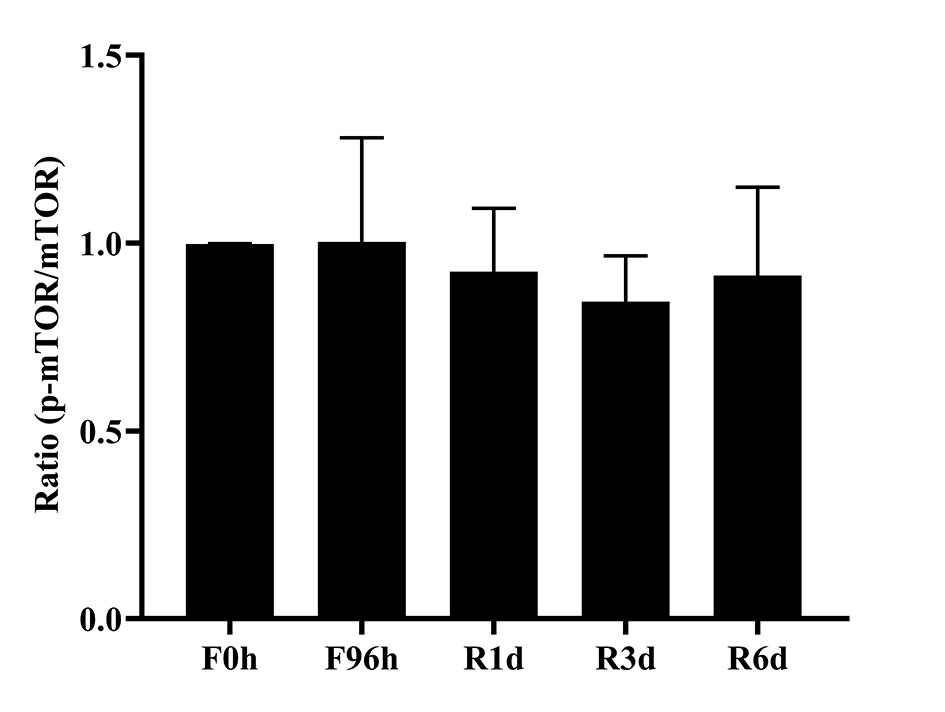

Supplement: Supplemental Information 1 [file peerj-10-14009-s001.zip › Raw data/Figure 7K.png]

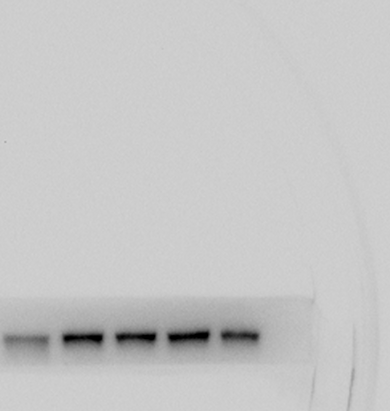

Supplement: Supplemental Information 1 [file peerj-10-14009-s001.zip › Raw data/Figure 7L SIRT1-1.png]

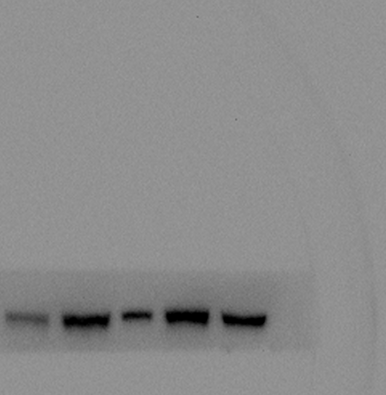

Supplement: Supplemental Information 1 [file peerj-10-14009-s001.zip › Raw data/Figure 7L SIRT1-2.png]

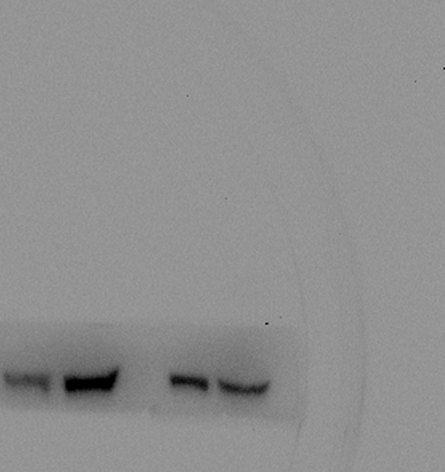

Supplement: Supplemental Information 1 [file peerj-10-14009-s001.zip › Raw data/Figure 7L SIRT1-3.png]

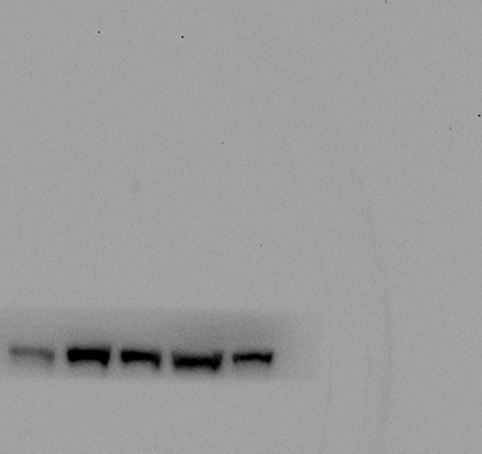

Supplement: Supplemental Information 1 [file peerj-10-14009-s001.zip › Raw data/Figure 7L SIRT1-4.png]

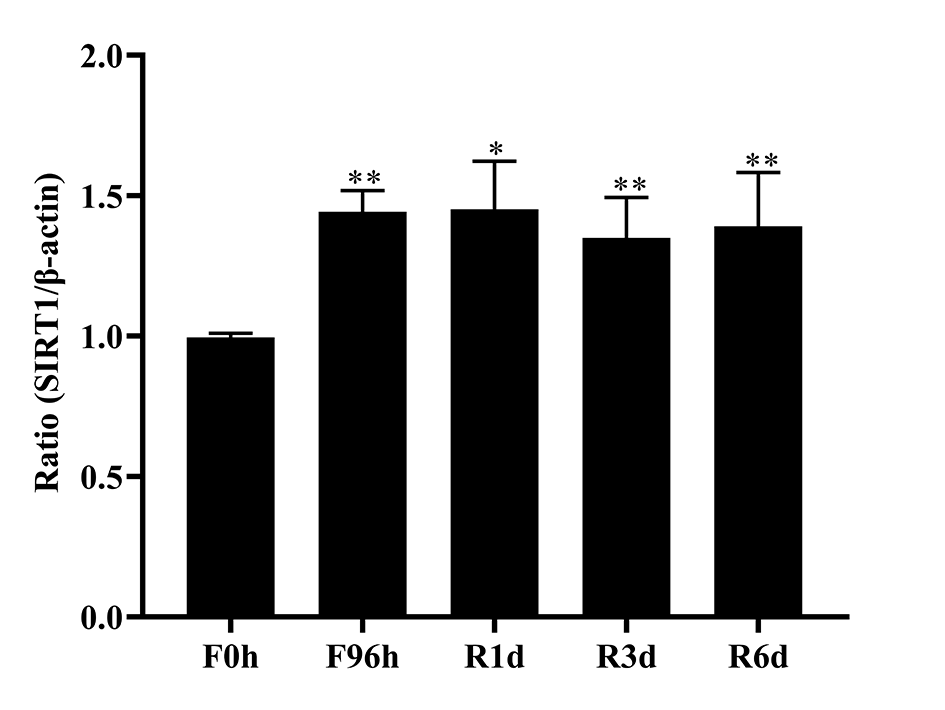

Supplement: Supplemental Information 1 [file peerj-10-14009-s001.zip › Raw data/Figure 7L.png]

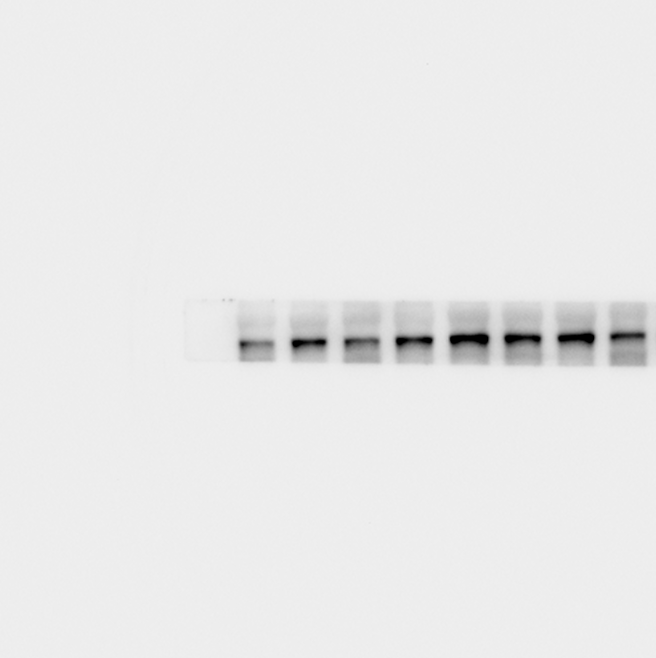

Supplement: Supplemental Information 1 [file peerj-10-14009-s001.zip › Raw data/Figure7C p-AMPK-1.png]

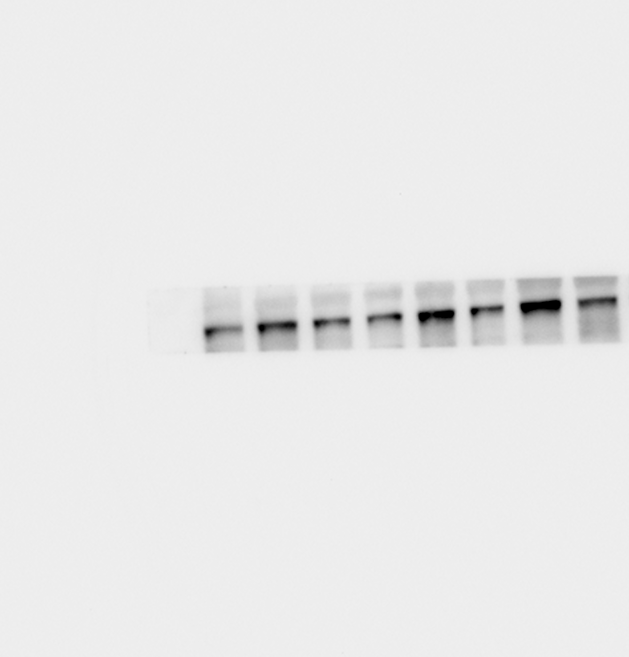

Supplement: Supplemental Information 1 [file peerj-10-14009-s001.zip › Raw data/Figure7C p-AMPK-2.png]

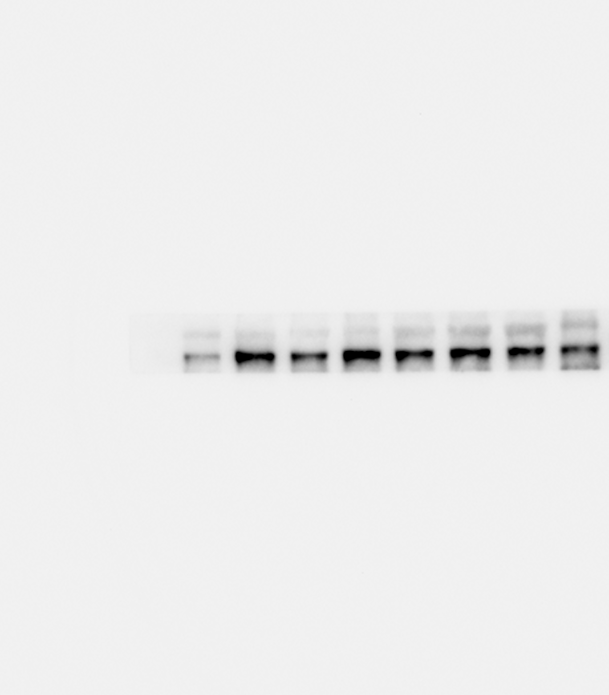

Supplement: Supplemental Information 1 [file peerj-10-14009-s001.zip › Raw data/Figure7C p-AMPK-3.png]

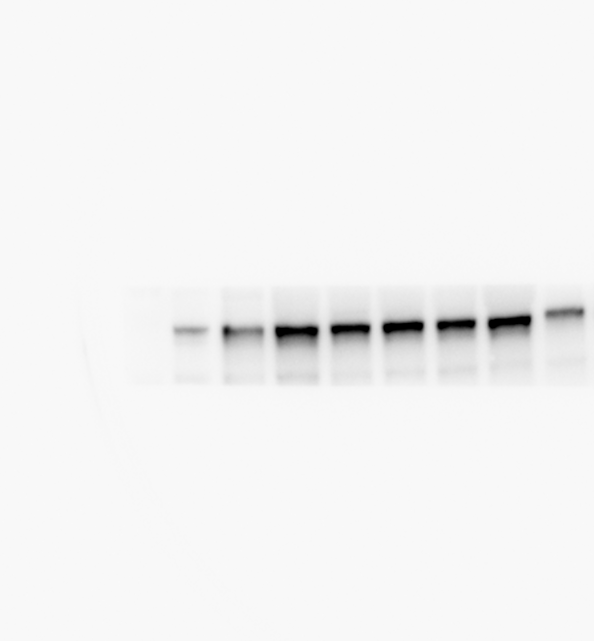

Supplement: Supplemental Information 1 [file peerj-10-14009-s001.zip › Raw data/Figure7C p-AMPK-4.png]

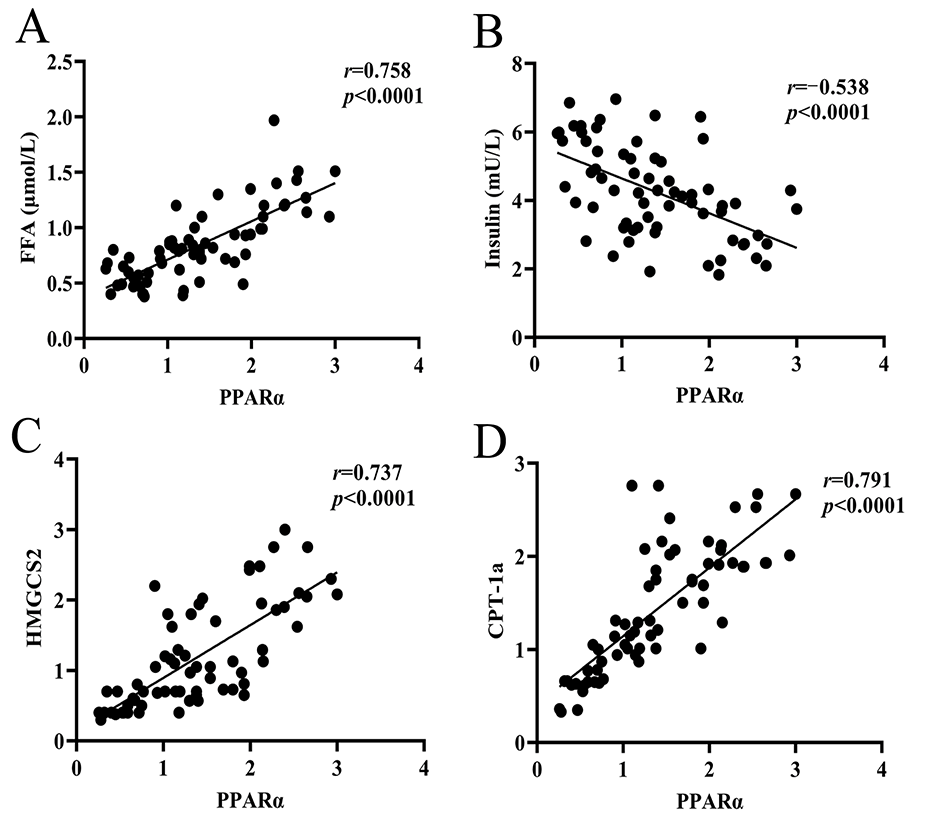

Supplement: Supplemental Information 2 [file peerj-10-14009-s002.zip › Supplement data/Figure S1.png]

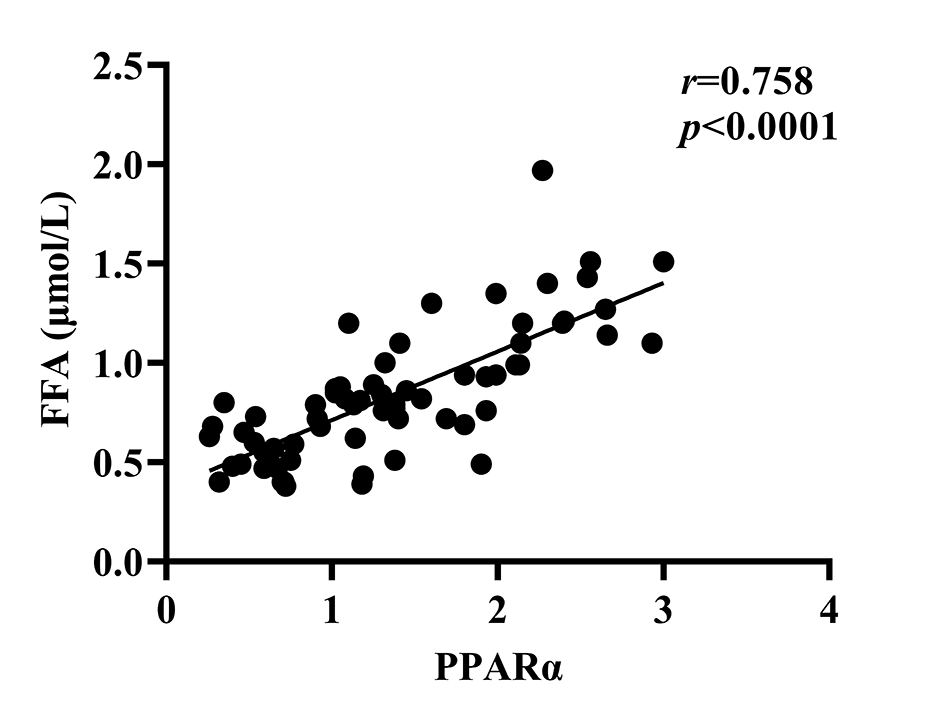

Supplement: Supplemental Information 2 [file peerj-10-14009-s002.zip › Supplement data/Figure S1A.png]

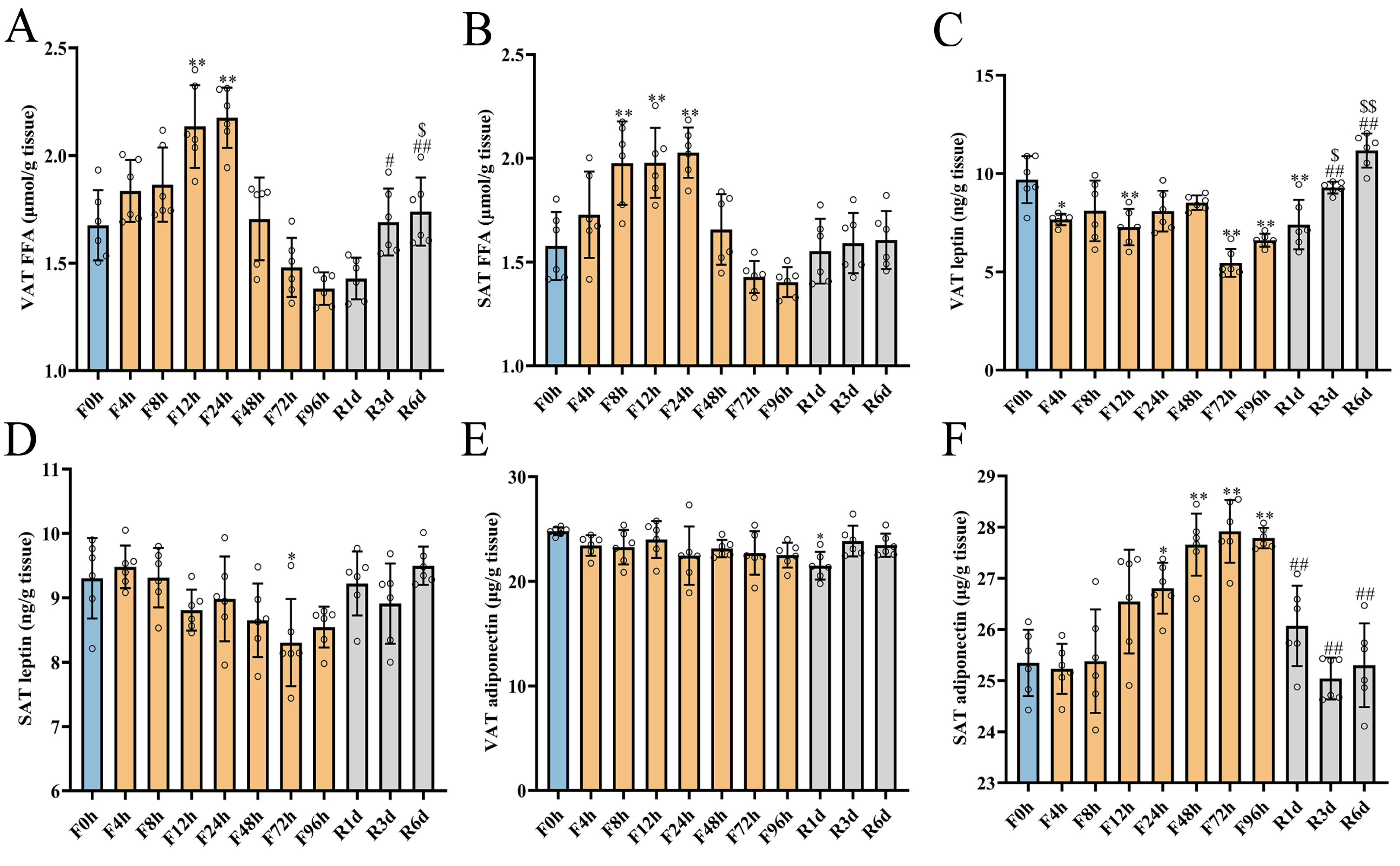

Supplement: Supplemental Information 2 [file peerj-10-14009-s002.zip › Supplement data/Figure S2.png]

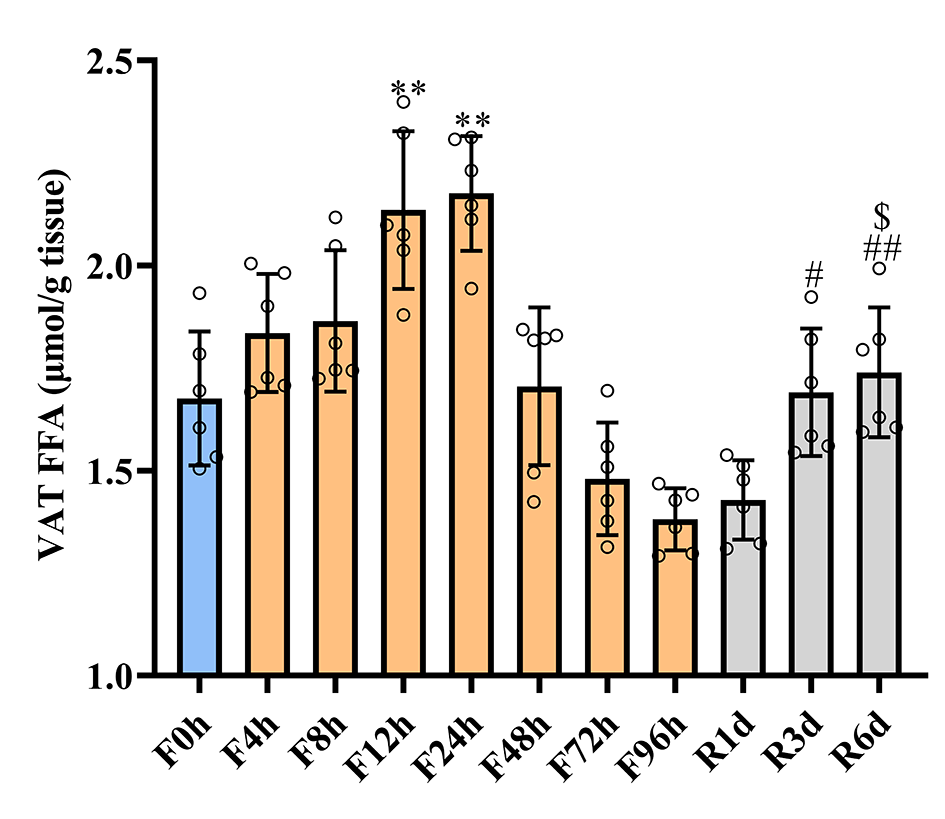

Supplement: Supplemental Information 2 [file peerj-10-14009-s002.zip › Supplement data/Figure S2A.png]

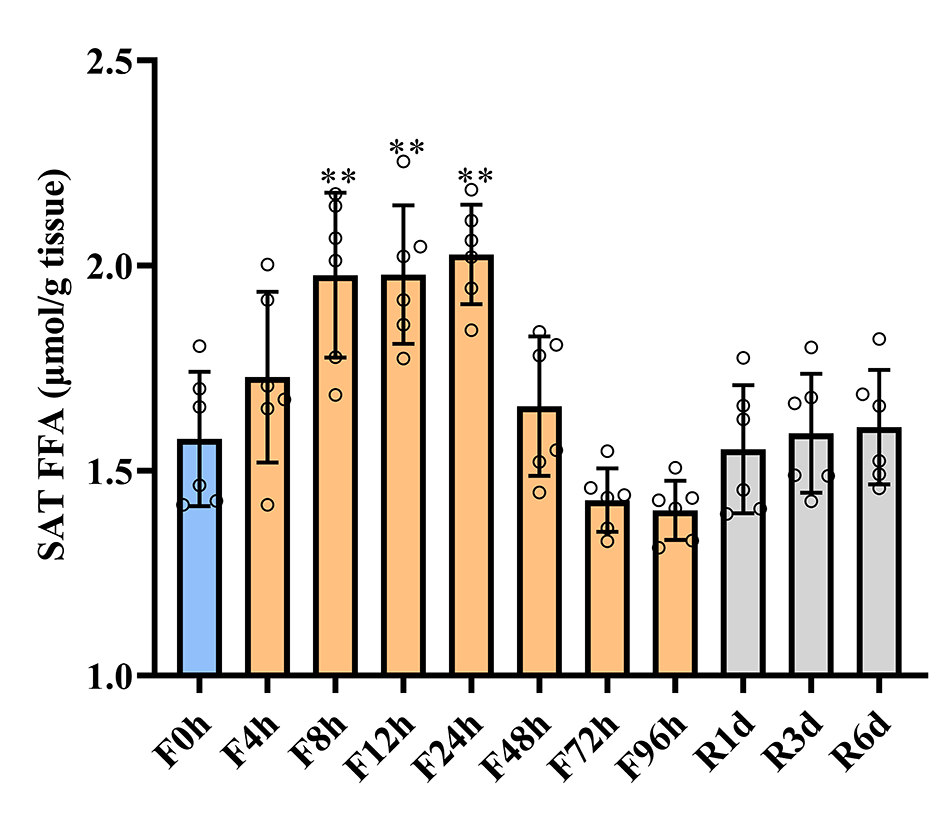

Supplement: Supplemental Information 2 [file peerj-10-14009-s002.zip › Supplement data/Figure S2B.png]

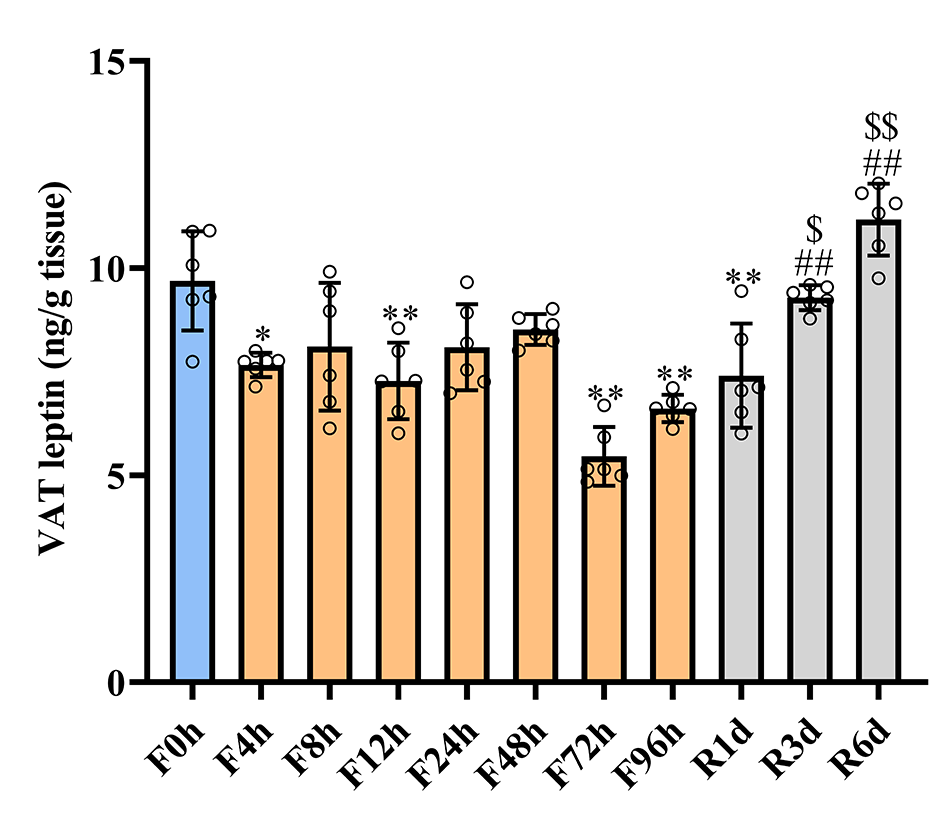

Supplement: Supplemental Information 2 [file peerj-10-14009-s002.zip › Supplement data/Figure S2C.png]

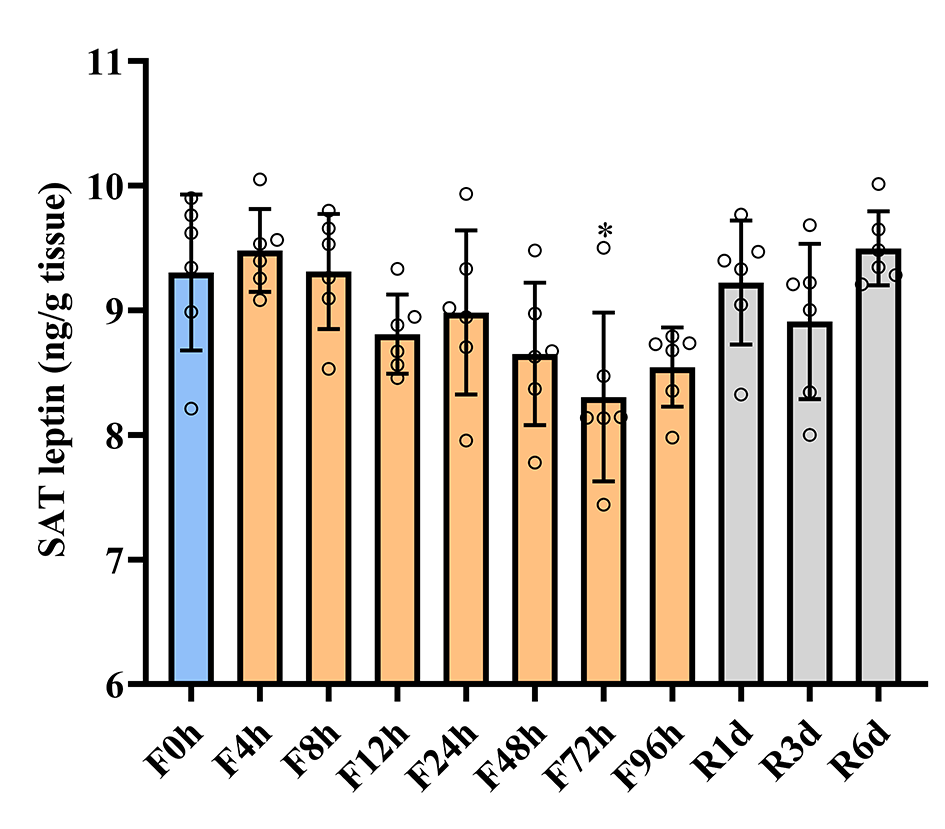

Supplement: Supplemental Information 2 [file peerj-10-14009-s002.zip › Supplement data/Figure S2D.png]

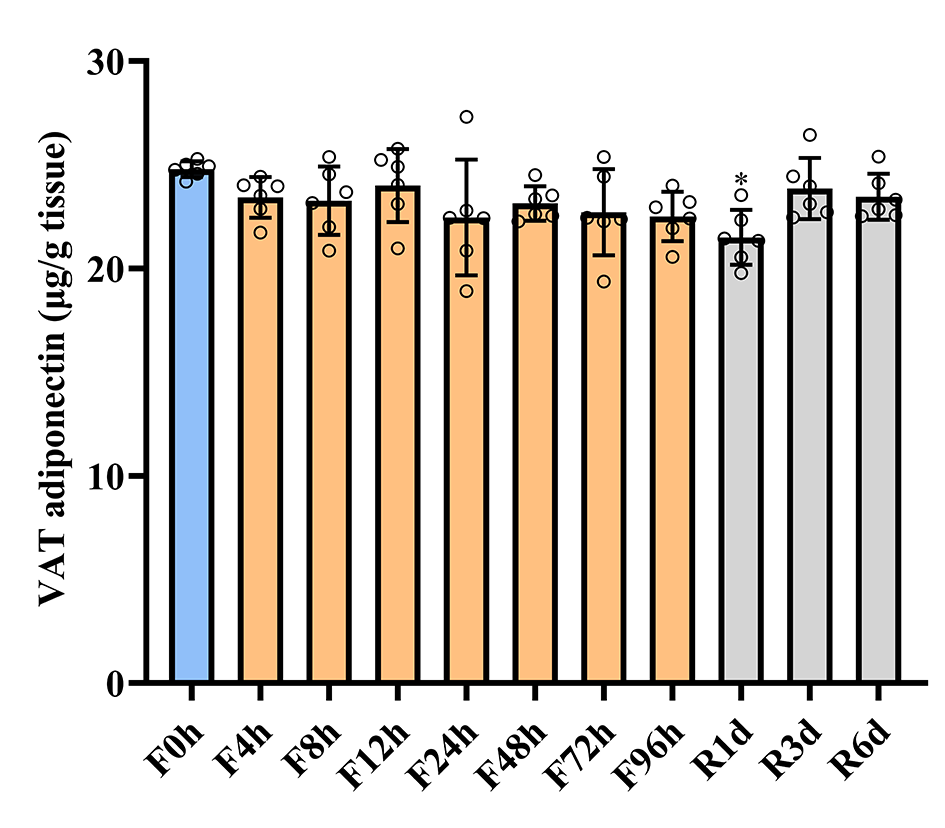

Supplement: Supplemental Information 2 [file peerj-10-14009-s002.zip › Supplement data/Figure S2E.png]

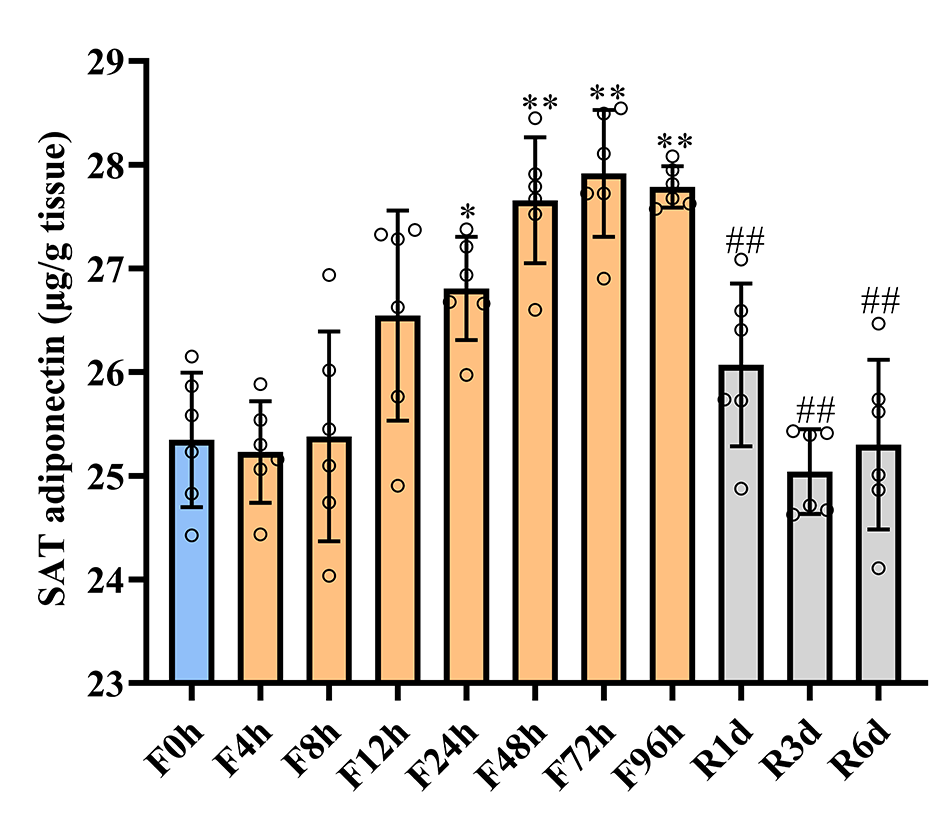

Supplement: Supplemental Information 2 [file peerj-10-14009-s002.zip › Supplement data/Figure S2F.png]

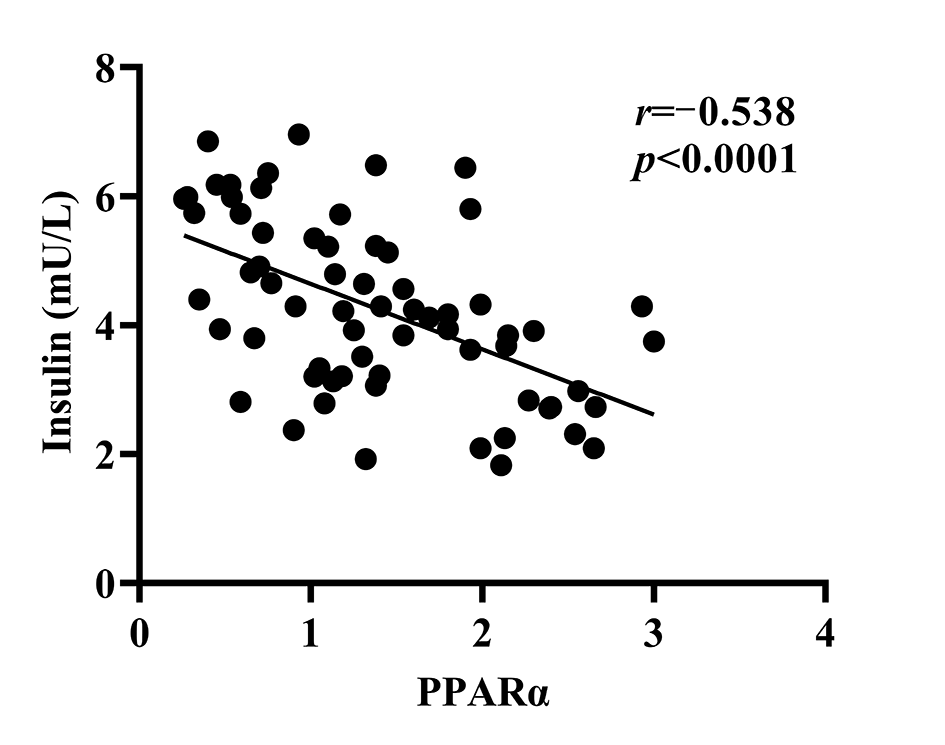

Supplement: Supplemental Information 2 [file peerj-10-14009-s002.zip › Supplement data/FigureS1B.png]

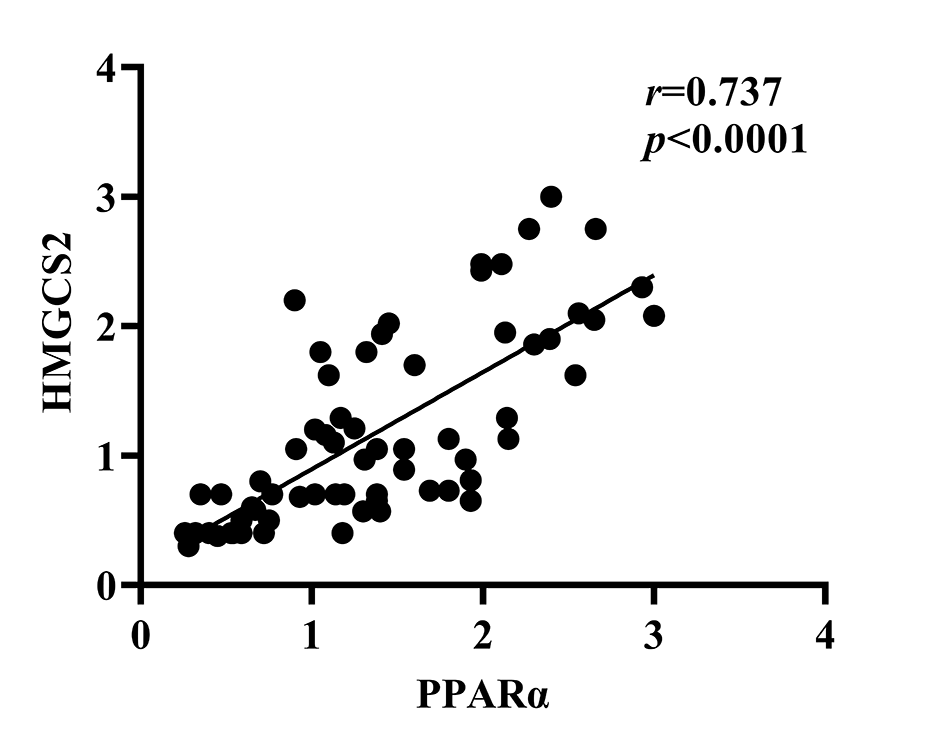

Supplement: Supplemental Information 2 [file peerj-10-14009-s002.zip › Supplement data/FigureS1C.png]

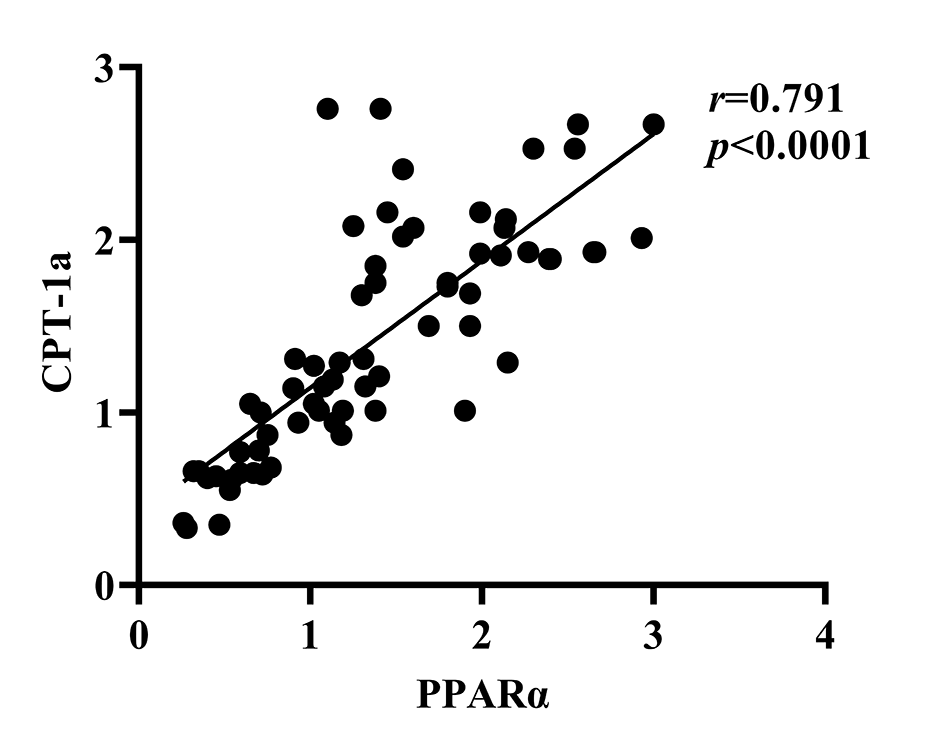

Supplement: Supplemental Information 2 [file peerj-10-14009-s002.zip › Supplement data/FigureS1D.png]

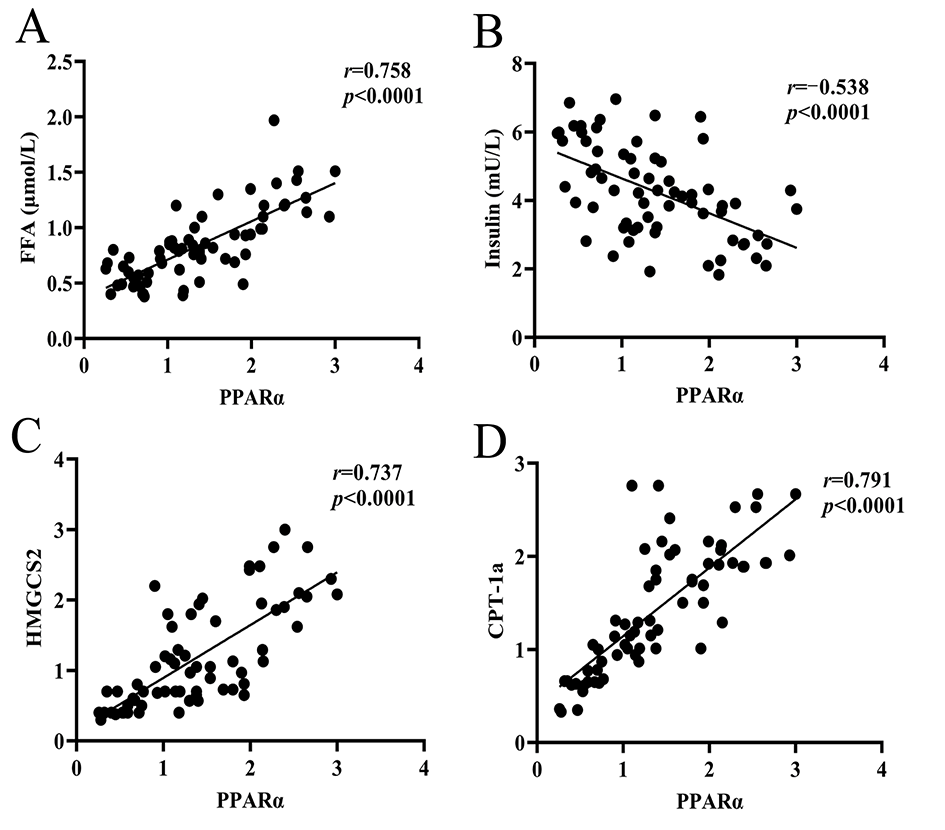

Supplement: Figure S1 — (A) FFA (µmmol/L); (B) insulin (mU/L); (C) the expression of HMGCS2 and (D) the expression of CPT-1a. [file peerj-10-14009-s003.png]

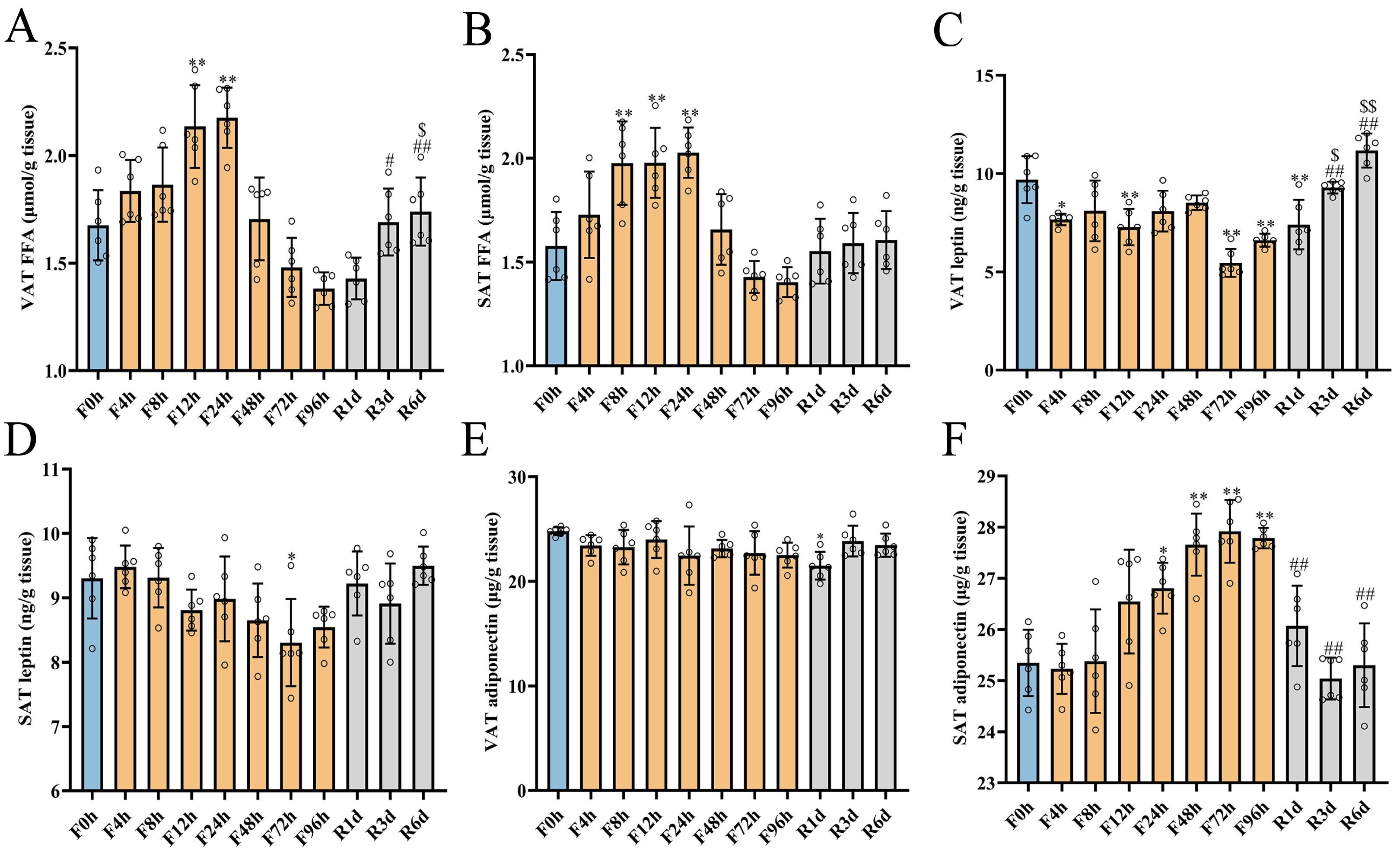

Supplement: Figure S2 — VAT: visceral adipose tissue. SAT: subcutaneous adipose tissue. (A) FFA in VAT; (B) FFA in SATs; (C) Leptin in VAT; (D) Leptin in SAT; (E) Adiponectin in VAT; (F) Adiponectin in SAT. [file peerj-10-14009-s004.png]
