# Supplementary figures and images for: Hepatic metabolite responses to 4-day complete fasting and subsequent refeeding in rats (part 1 of 2)
Source: PeerJ. 2022 Sep 20;10:e14009. doi: 10.7717/peerj.14009 (PMC9504452; doi:10.7717/peerj.14009)

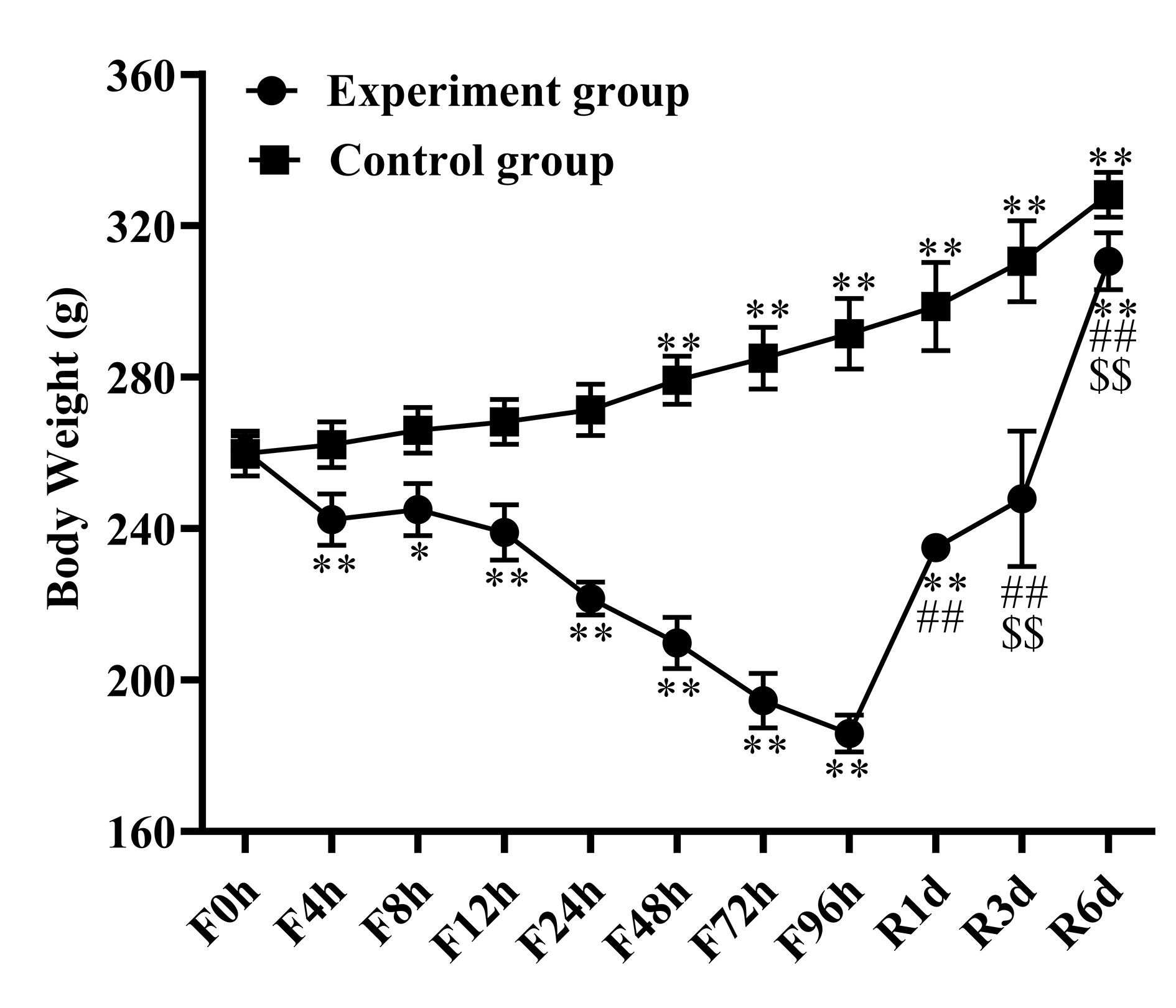

Supplement: Supplemental Information 1 [file peerj-10-14009-s001.zip › Raw data/Figure 1.png]

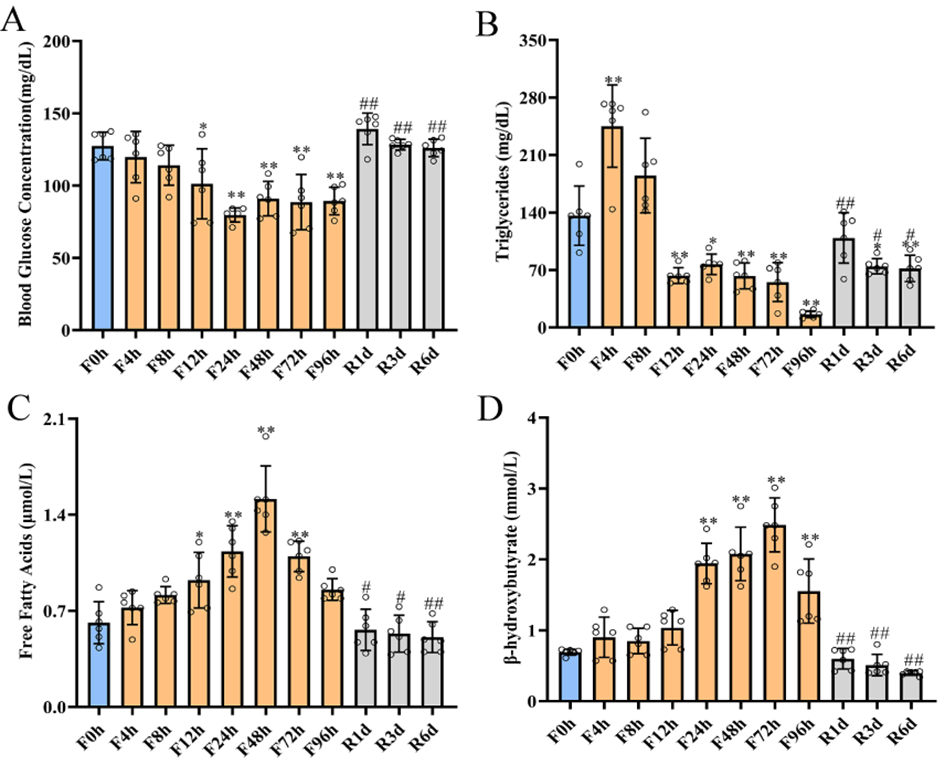

Supplement: Supplemental Information 1 [file peerj-10-14009-s001.zip › Raw data/Figure 2.png]

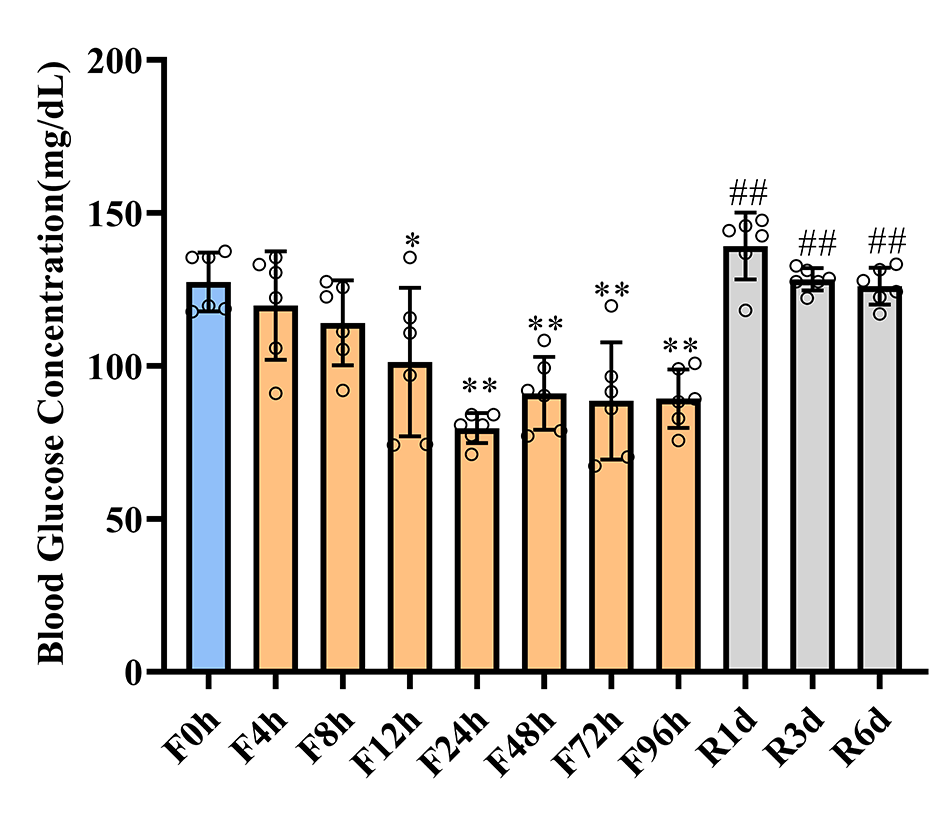

Supplement: Supplemental Information 1 [file peerj-10-14009-s001.zip › Raw data/Figure 2A.png]

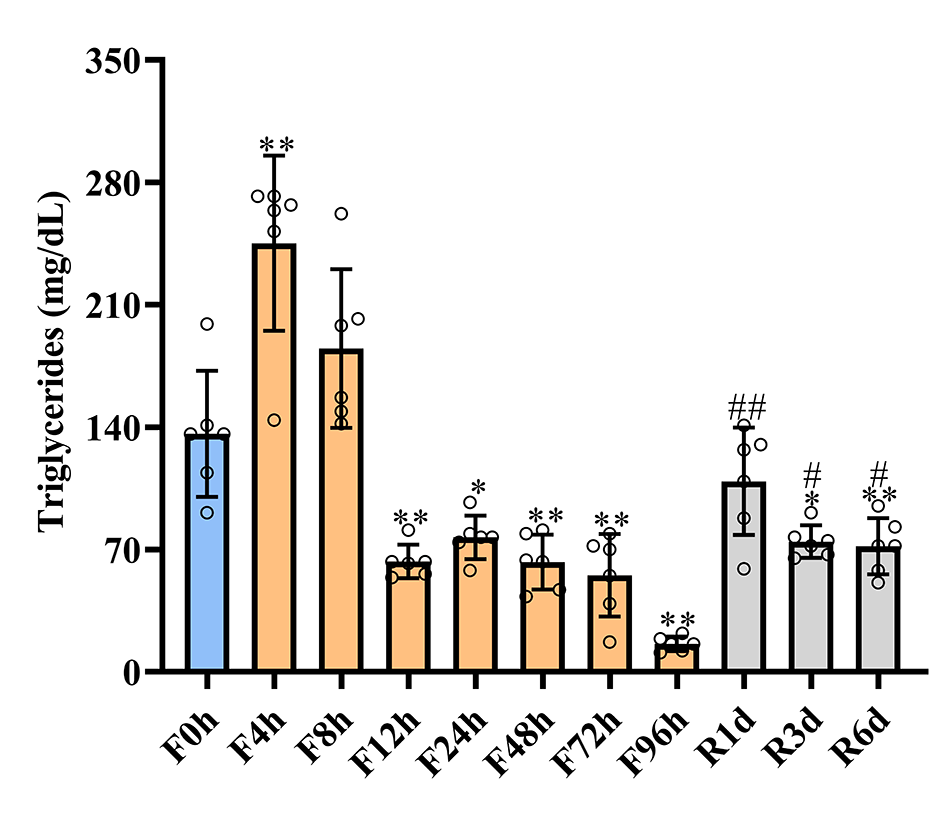

Supplement: Supplemental Information 1 [file peerj-10-14009-s001.zip › Raw data/Figure 2B.png]

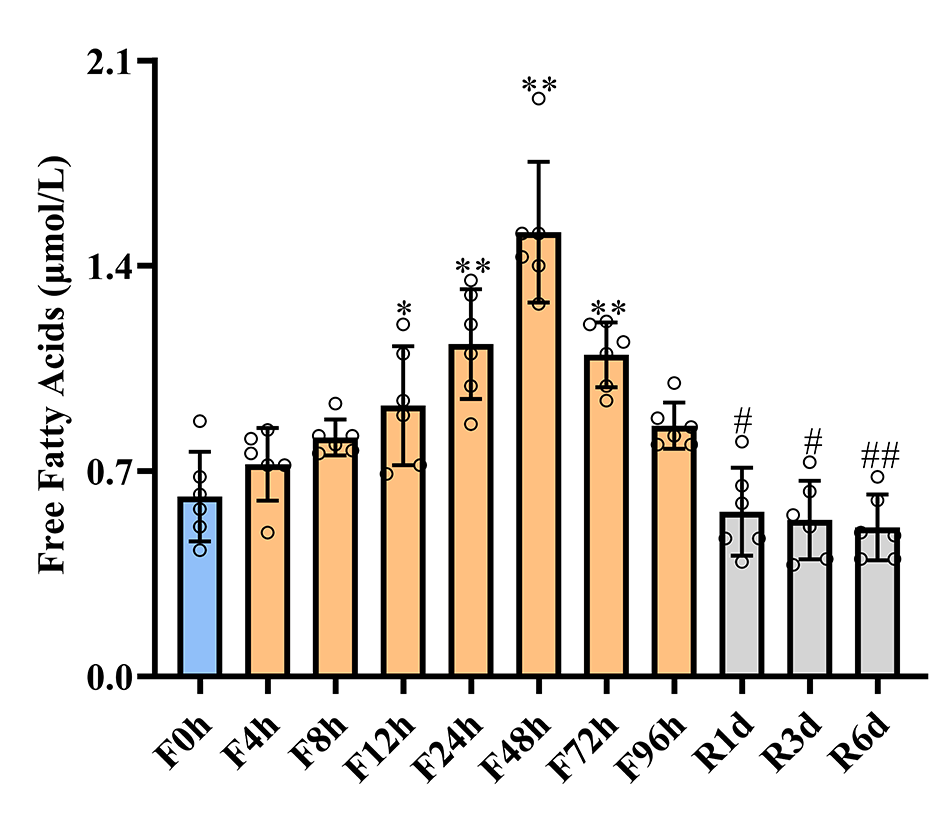

Supplement: Supplemental Information 1 [file peerj-10-14009-s001.zip › Raw data/Figure 2C.png]

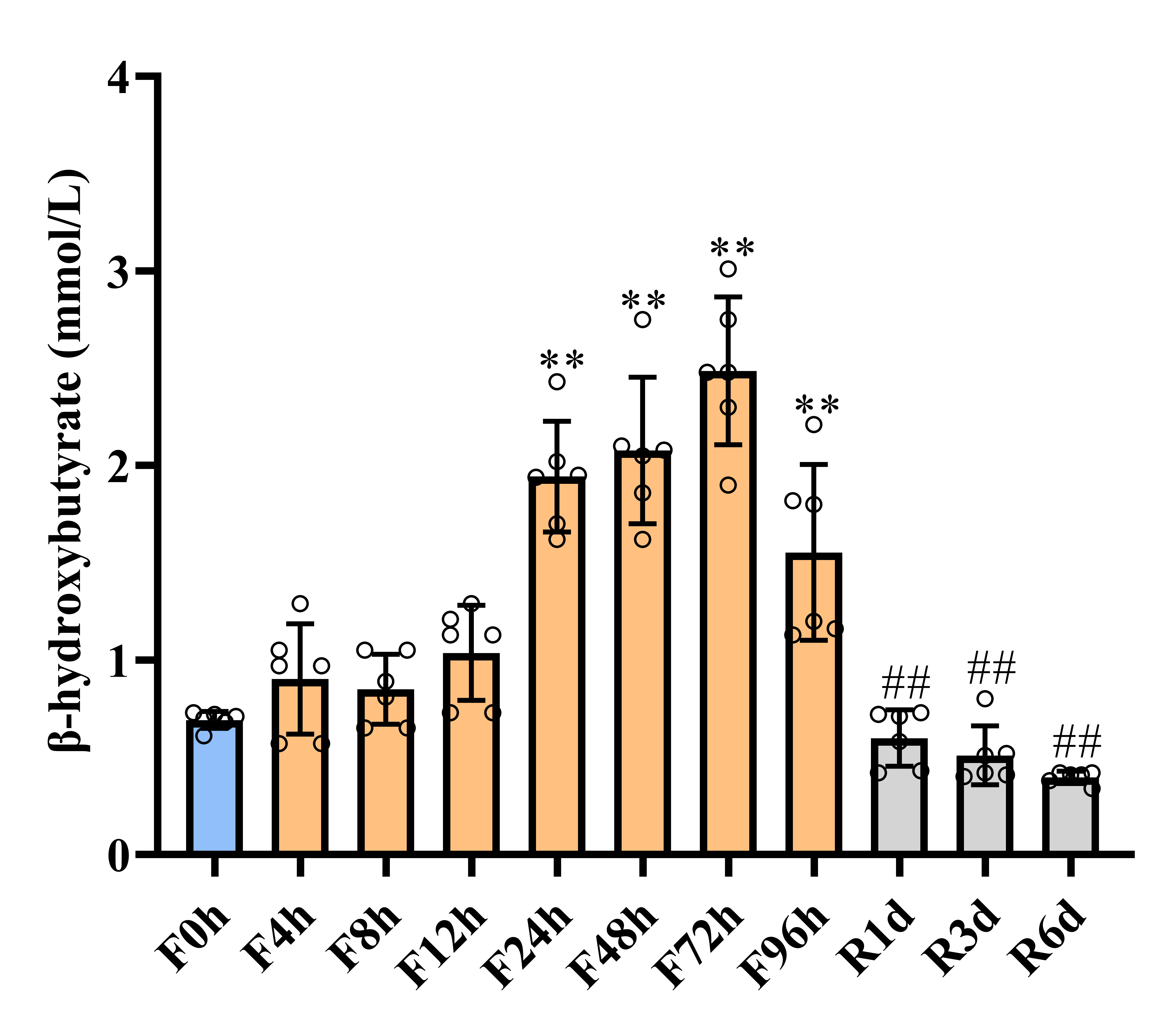

Supplement: Supplemental Information 1 [file peerj-10-14009-s001.zip › Raw data/Figure 2D.png]

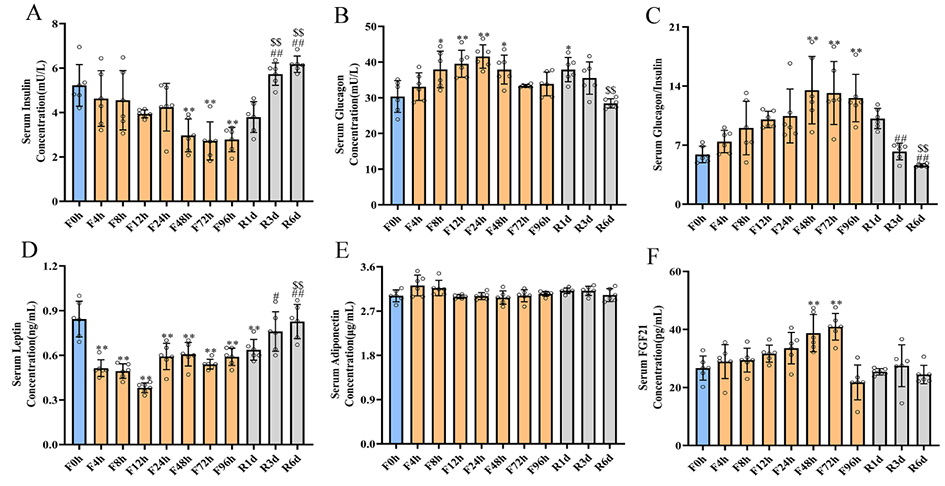

Supplement: Supplemental Information 1 [file peerj-10-14009-s001.zip › Raw data/Figure 3.png]

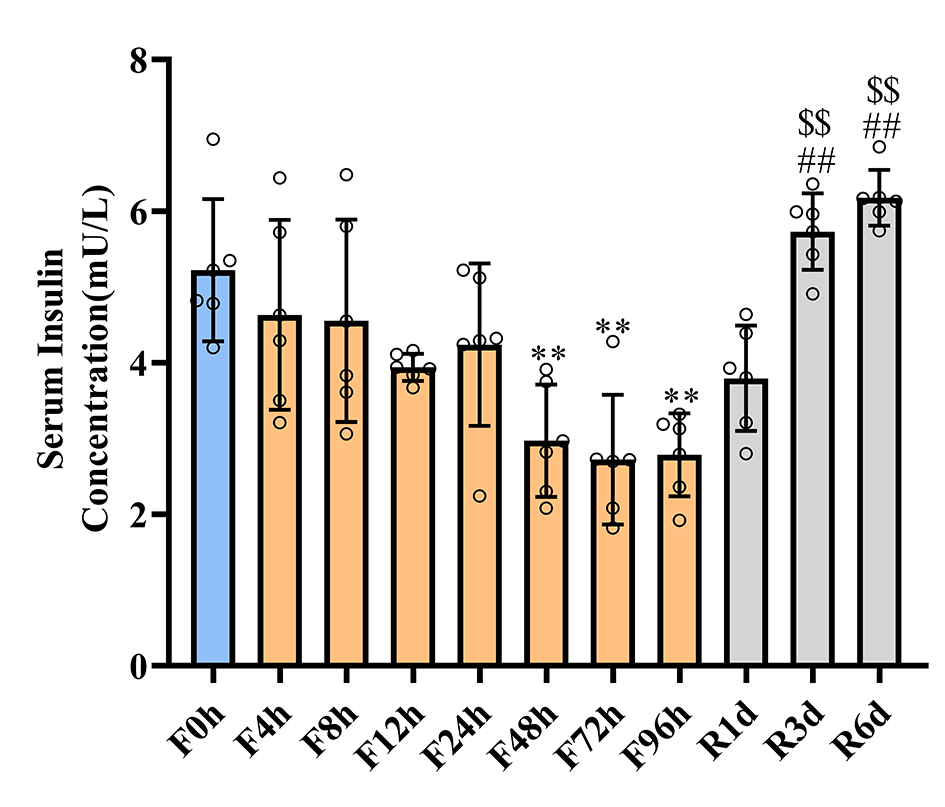

Supplement: Supplemental Information 1 [file peerj-10-14009-s001.zip › Raw data/Figure 3A.png]

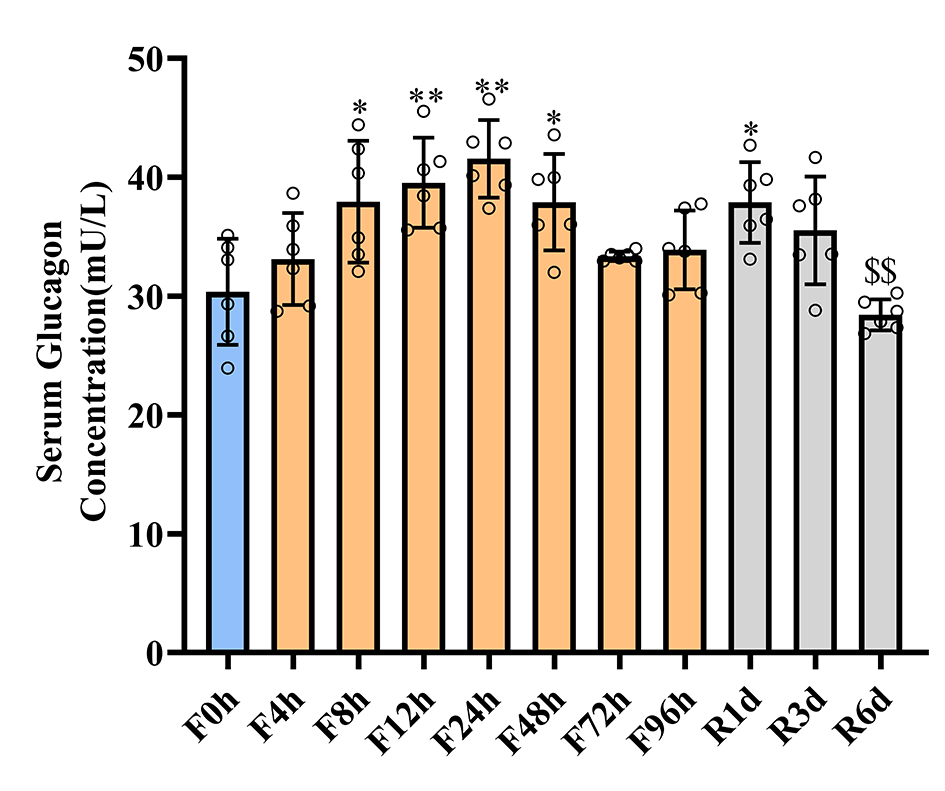

Supplement: Supplemental Information 1 [file peerj-10-14009-s001.zip › Raw data/Figure 3B.png]

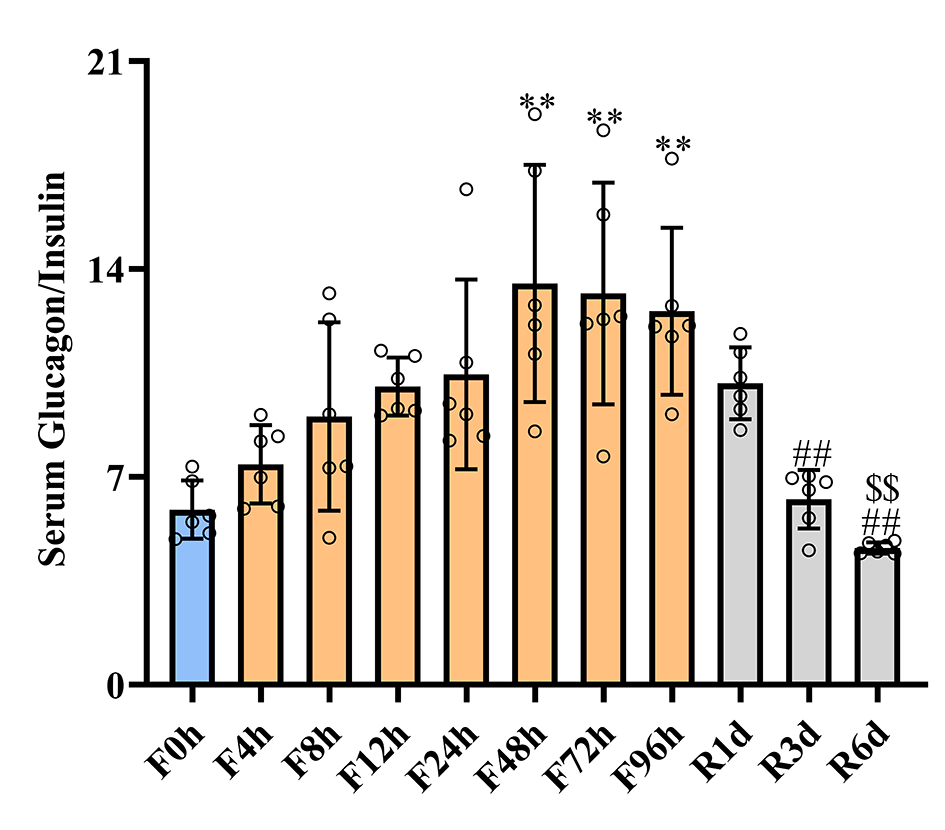

Supplement: Supplemental Information 1 [file peerj-10-14009-s001.zip › Raw data/Figure 3C.png]

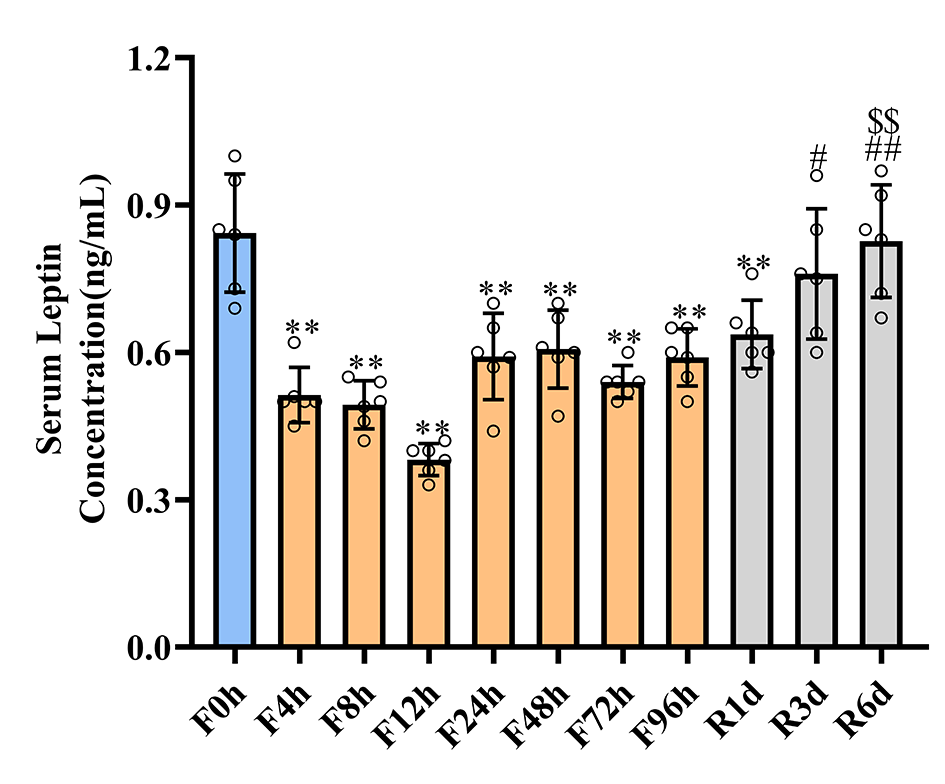

Supplement: Supplemental Information 1 [file peerj-10-14009-s001.zip › Raw data/Figure 3D.png]

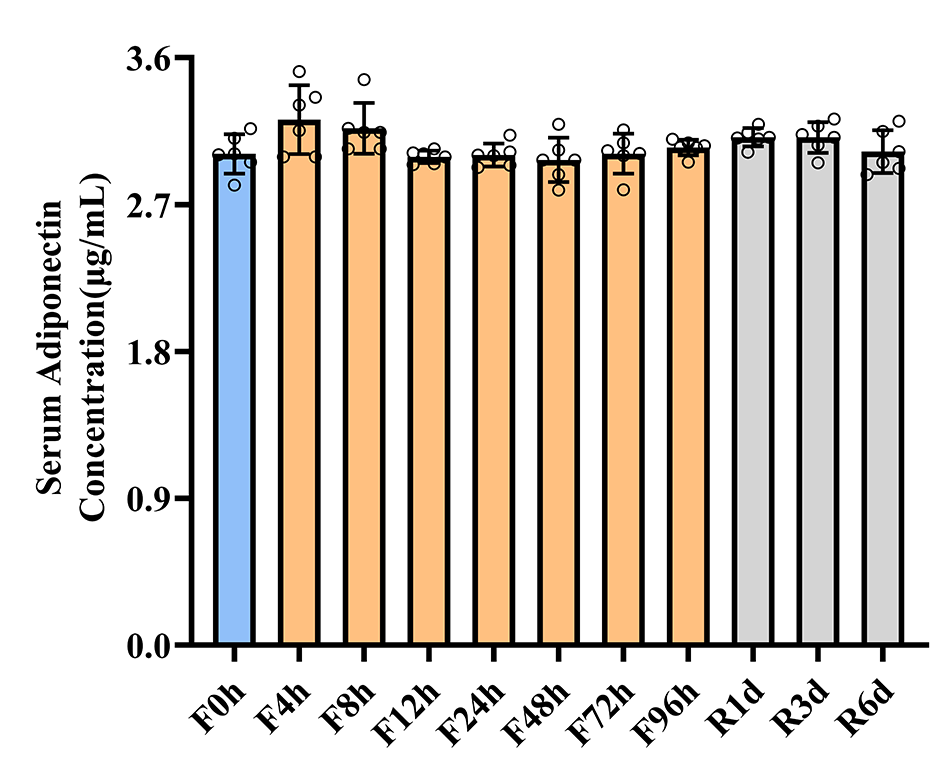

Supplement: Supplemental Information 1 [file peerj-10-14009-s001.zip › Raw data/Figure 3E.png]

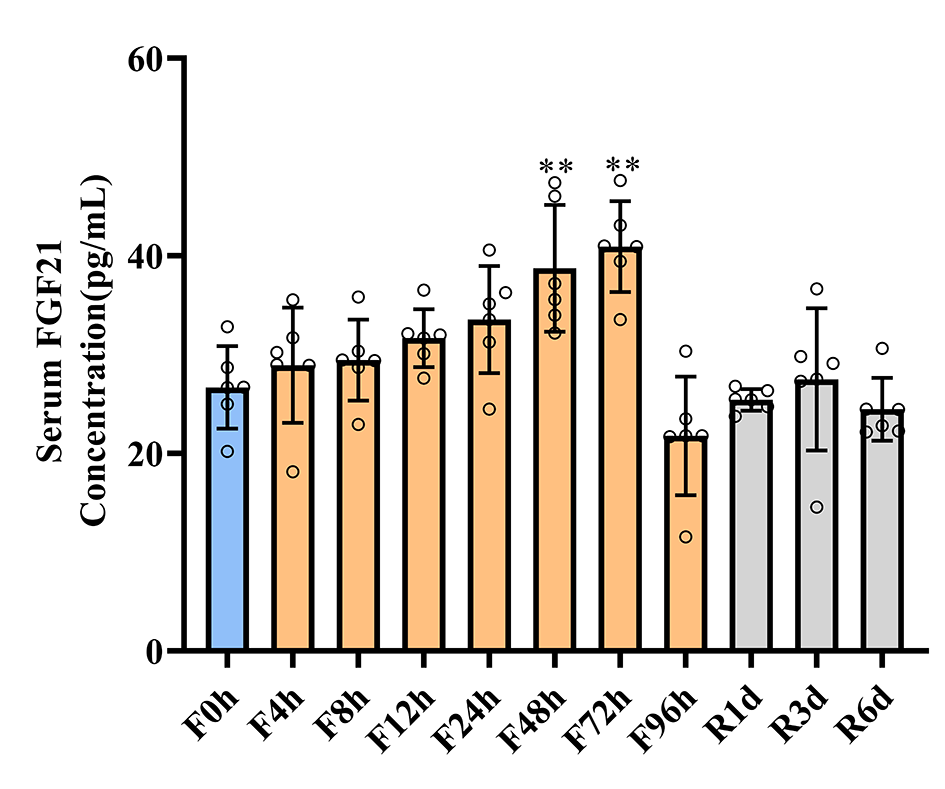

Supplement: Supplemental Information 1 [file peerj-10-14009-s001.zip › Raw data/Figure 3F.png]

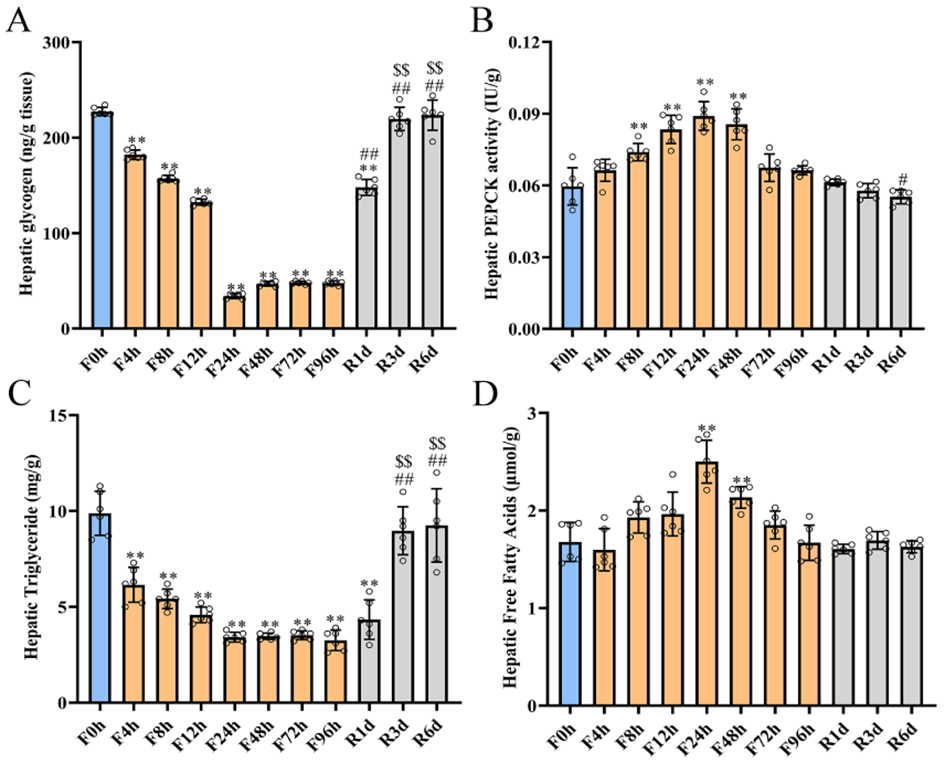

Supplement: Supplemental Information 1 [file peerj-10-14009-s001.zip › Raw data/Figure 4.png]

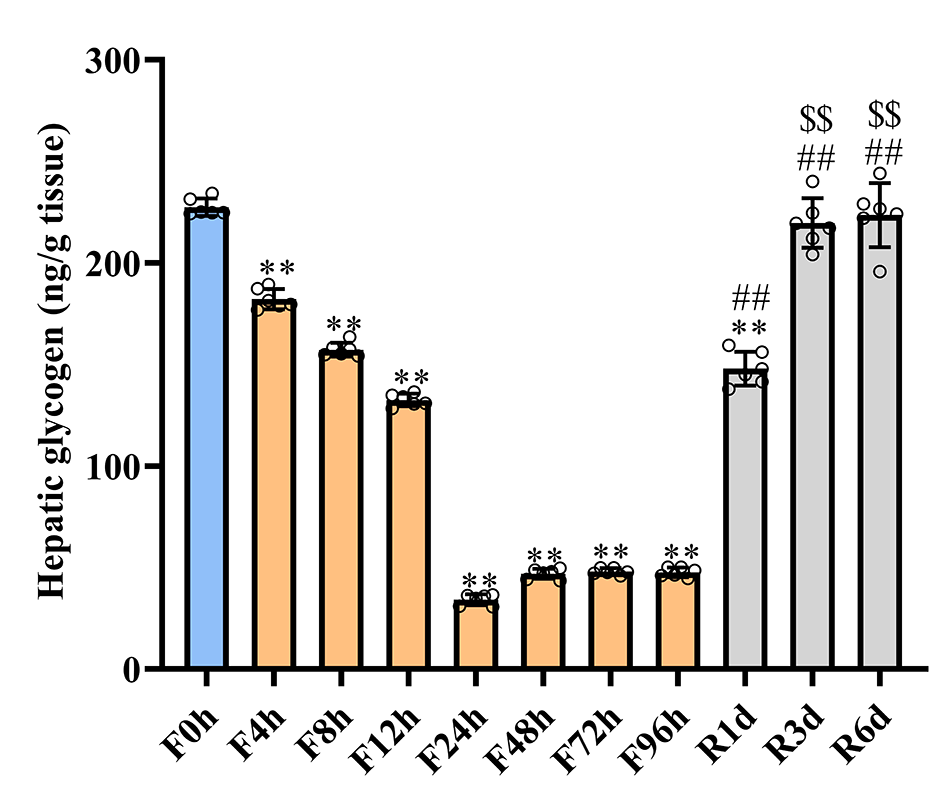

Supplement: Supplemental Information 1 [file peerj-10-14009-s001.zip › Raw data/Figure 4A.png]

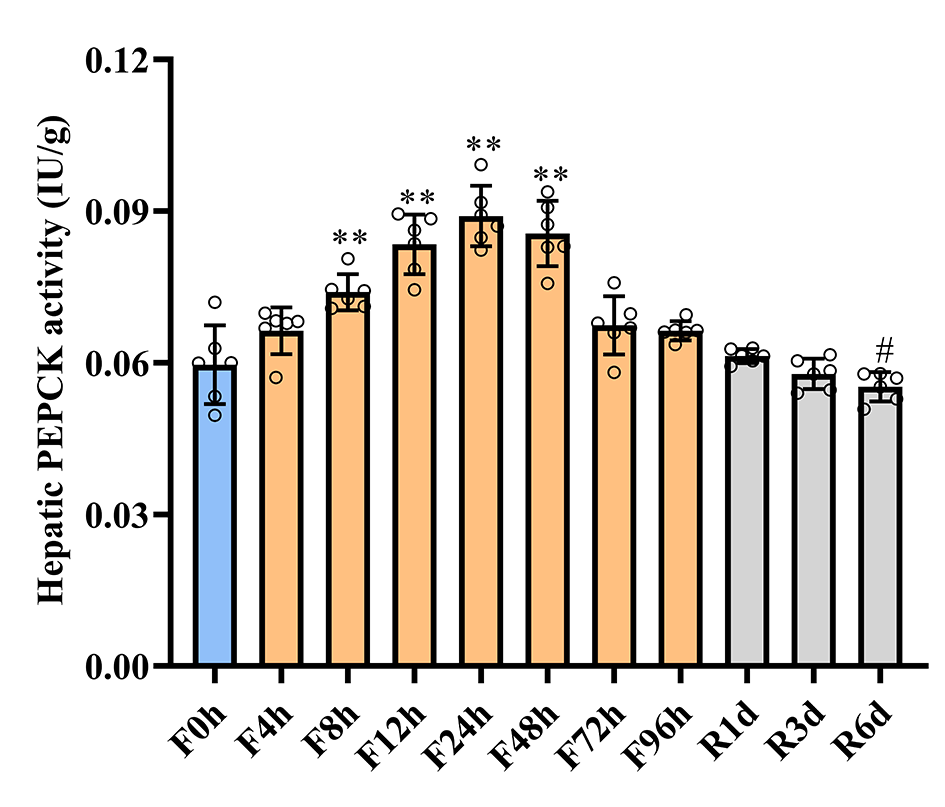

Supplement: Supplemental Information 1 [file peerj-10-14009-s001.zip › Raw data/Figure 4B.png]

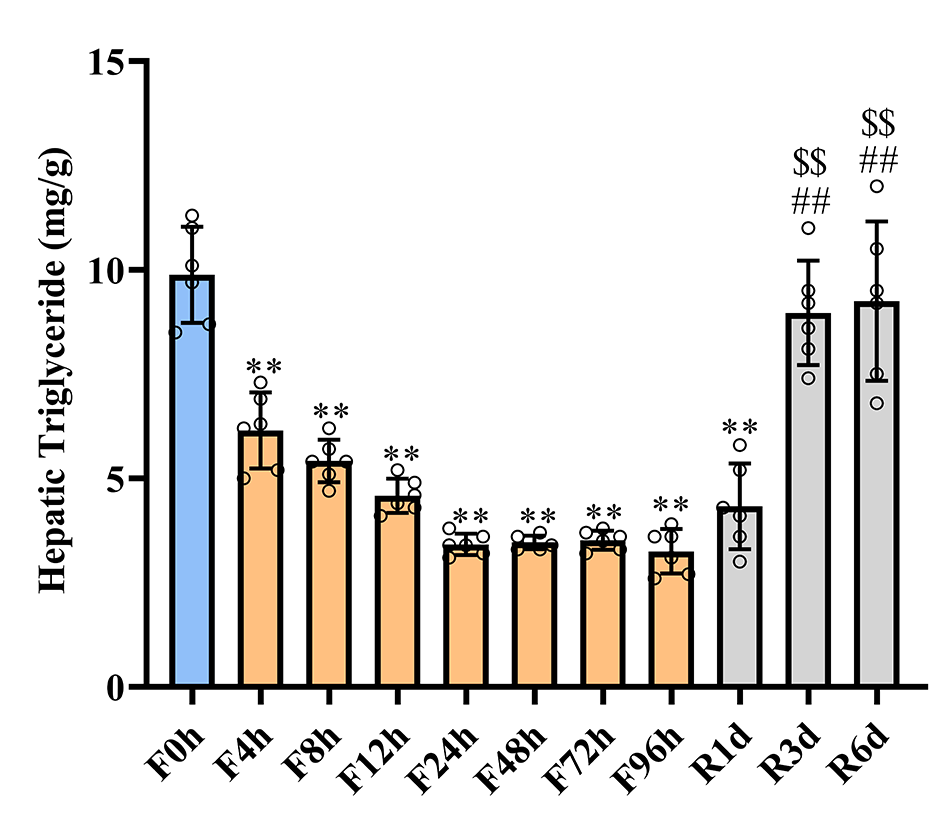

Supplement: Supplemental Information 1 [file peerj-10-14009-s001.zip › Raw data/Figure 4C.png]

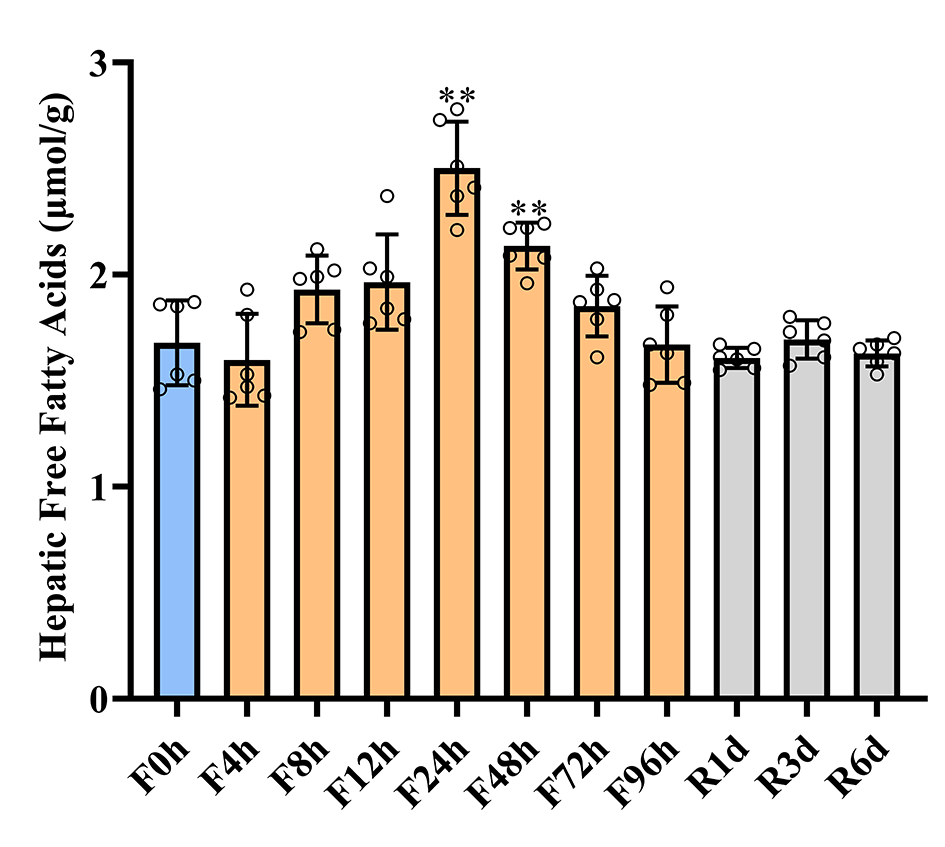

Supplement: Supplemental Information 1 [file peerj-10-14009-s001.zip › Raw data/Figure 4D.png]

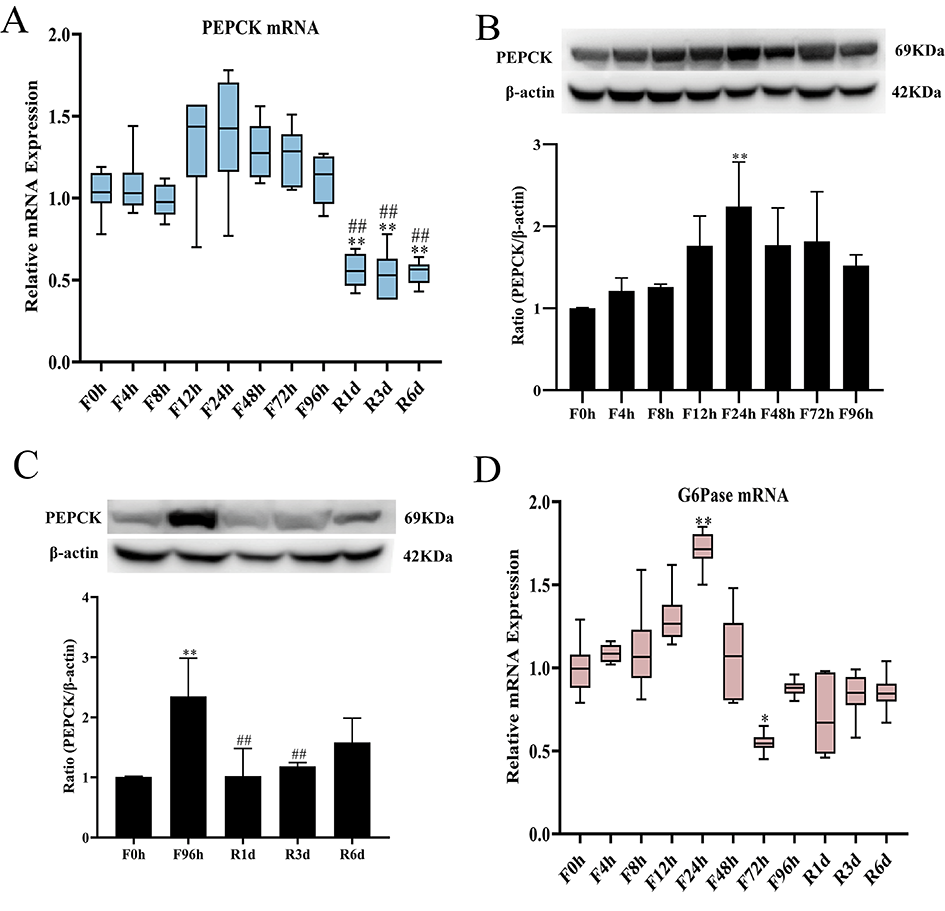

Supplement: Supplemental Information 1 [file peerj-10-14009-s001.zip › Raw data/Figure 5.png]

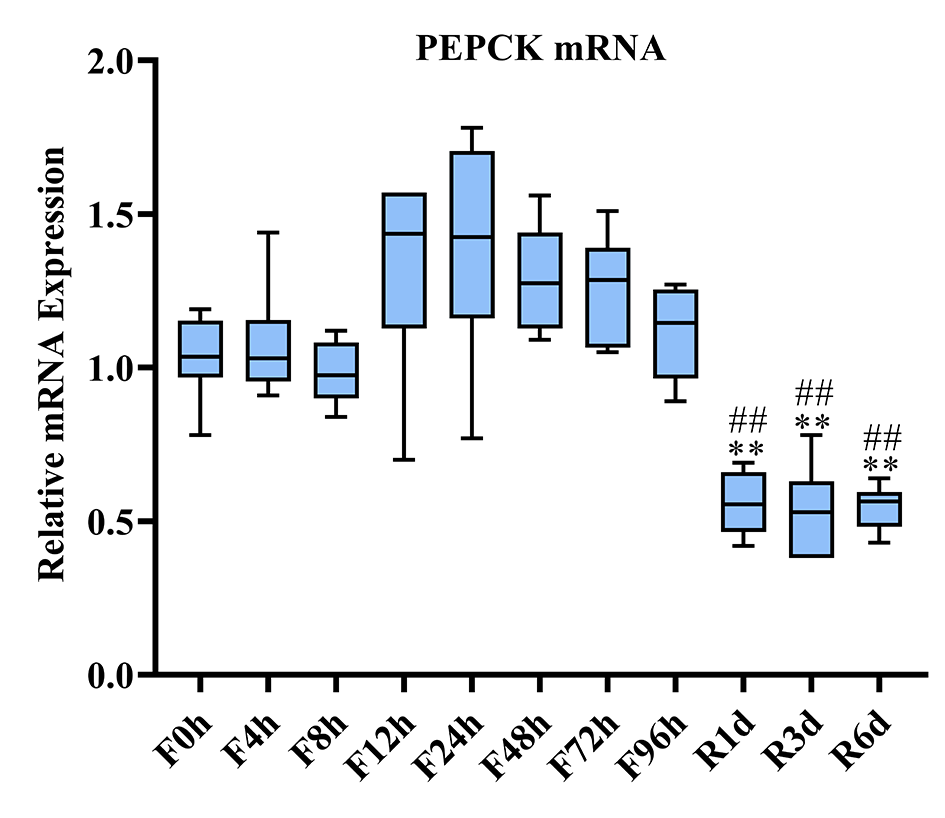

Supplement: Supplemental Information 1 [file peerj-10-14009-s001.zip › Raw data/Figure 5A.png]

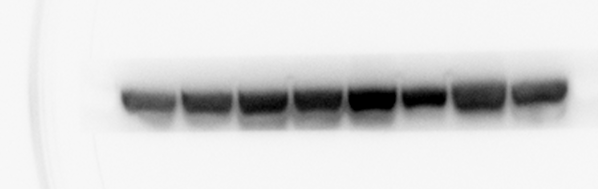

Supplement: Supplemental Information 1 [file peerj-10-14009-s001.zip › Raw data/Figure 5B PEPCK-1.png]

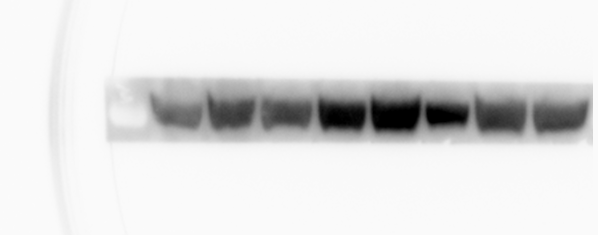

Supplement: Supplemental Information 1 [file peerj-10-14009-s001.zip › Raw data/Figure 5B PEPCK-2.png]

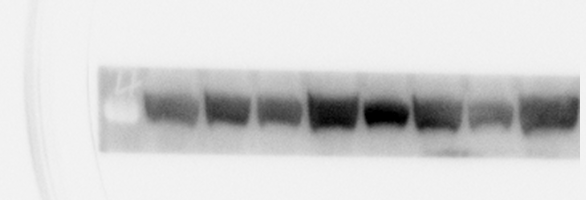

Supplement: Supplemental Information 1 [file peerj-10-14009-s001.zip › Raw data/Figure 5B PEPCK-3.png]

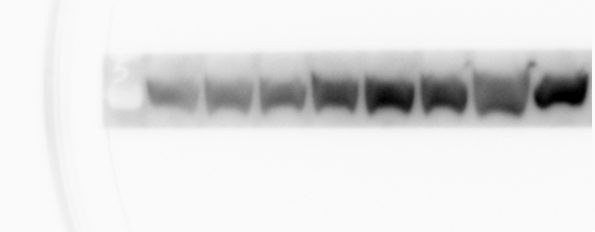

Supplement: Supplemental Information 1 [file peerj-10-14009-s001.zip › Raw data/Figure 5B PEPCK-4.png]

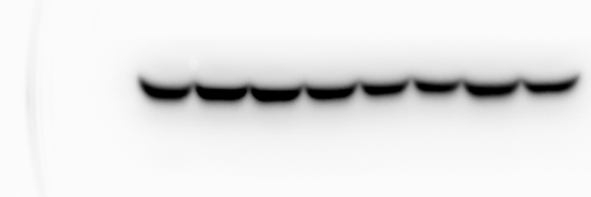

Supplement: Supplemental Information 1 [file peerj-10-14009-s001.zip › Raw data/Figure 5B a┬-actin-1.png]

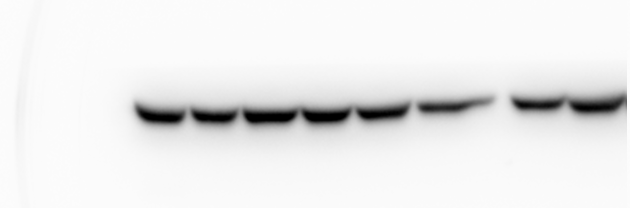

Supplement: Supplemental Information 1 [file peerj-10-14009-s001.zip › Raw data/Figure 5B a┬-actin-2.png]

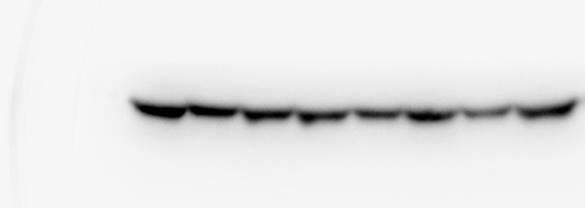

Supplement: Supplemental Information 1 [file peerj-10-14009-s001.zip › Raw data/Figure 5B a┬-actin-3.png]

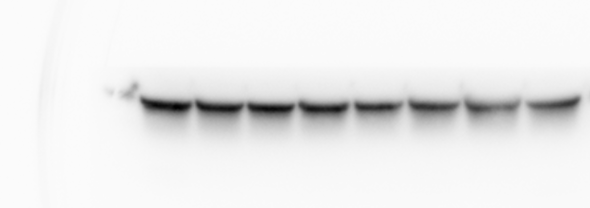

Supplement: Supplemental Information 1 [file peerj-10-14009-s001.zip › Raw data/Figure 5B a┬-actin-4.png]

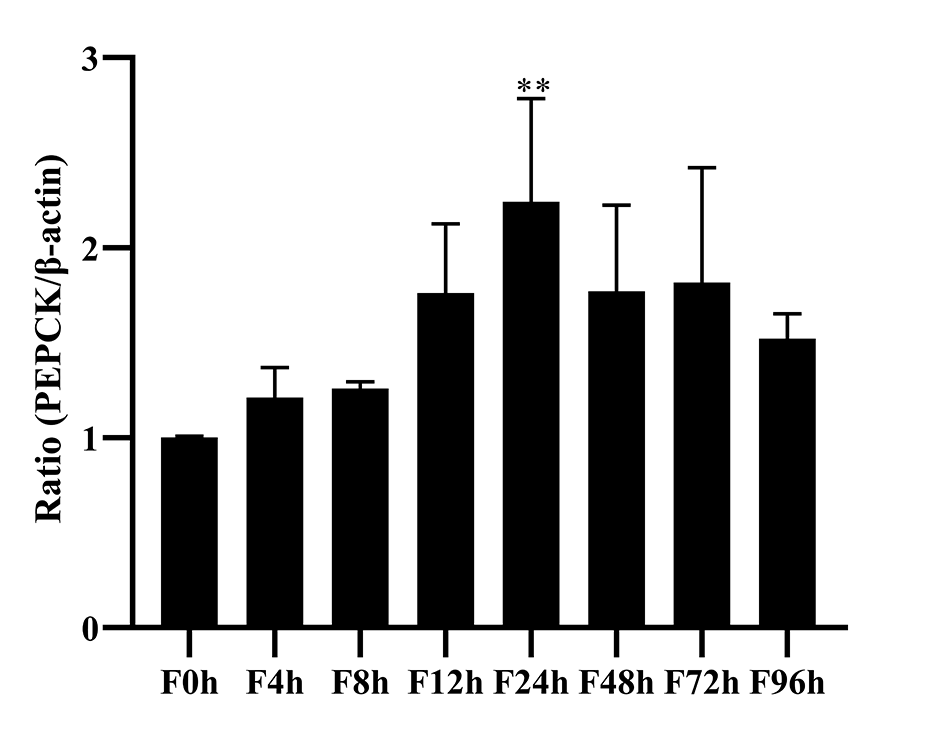

Supplement: Supplemental Information 1 [file peerj-10-14009-s001.zip › Raw data/Figure 5B.png]

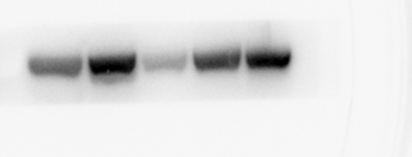

Supplement: Supplemental Information 1 [file peerj-10-14009-s001.zip › Raw data/Figure 5C PEPCK-1.png]

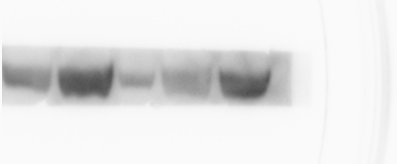

Supplement: Supplemental Information 1 [file peerj-10-14009-s001.zip › Raw data/Figure 5C PEPCK-2.png]

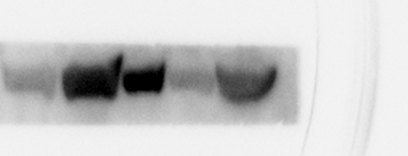

Supplement: Supplemental Information 1 [file peerj-10-14009-s001.zip › Raw data/Figure 5C PEPCK-3.png]

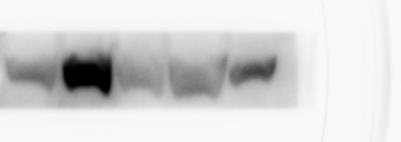

Supplement: Supplemental Information 1 [file peerj-10-14009-s001.zip › Raw data/Figure 5C PEPCK-4.png]

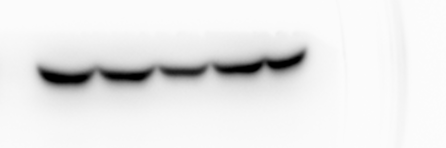

Supplement: Supplemental Information 1 [file peerj-10-14009-s001.zip › Raw data/Figure 5C a┬-actin-1.png]

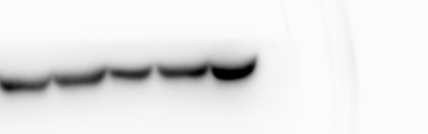

Supplement: Supplemental Information 1 [file peerj-10-14009-s001.zip › Raw data/Figure 5C a┬actin-2.png]

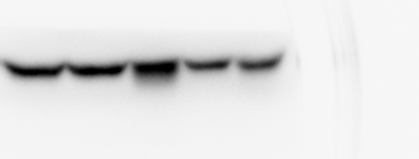

Supplement: Supplemental Information 1 [file peerj-10-14009-s001.zip › Raw data/Figure 5C a┬-actin-3.png]

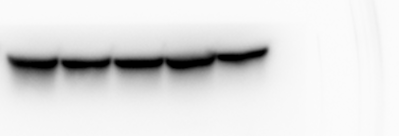

Supplement: Supplemental Information 1 [file peerj-10-14009-s001.zip › Raw data/Figure 5C a┬-actin-4.png]

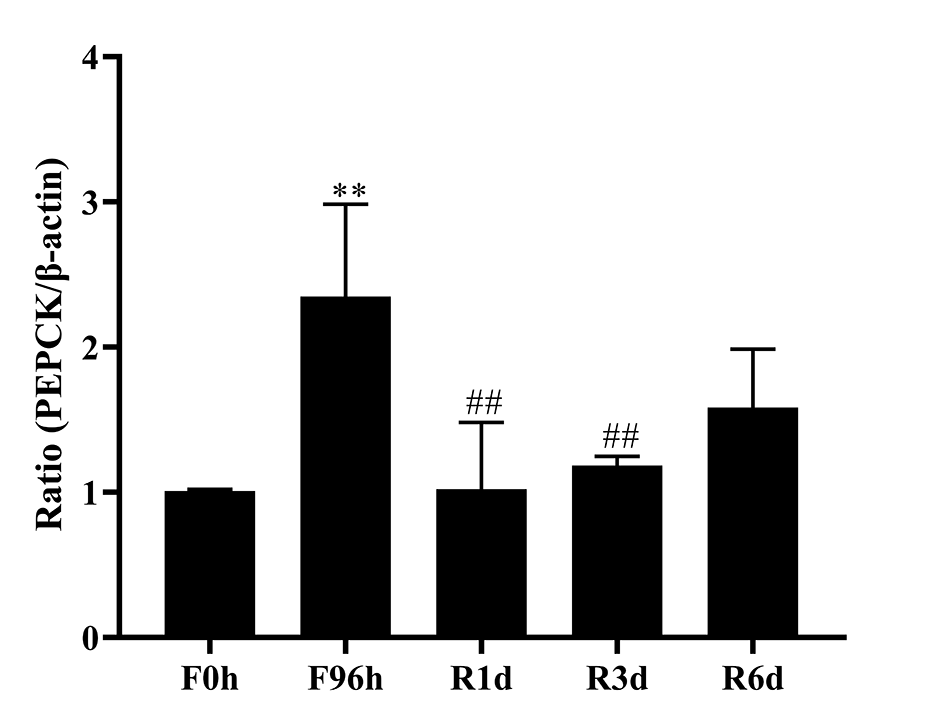

Supplement: Supplemental Information 1 [file peerj-10-14009-s001.zip › Raw data/Figure 5C.png]

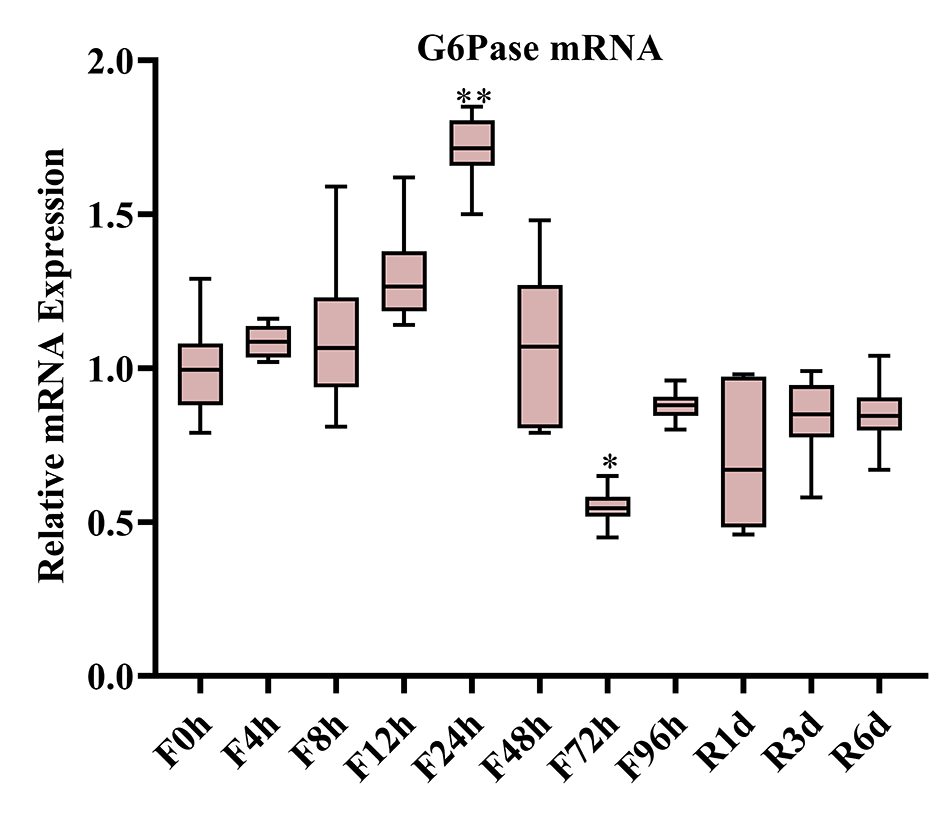

Supplement: Supplemental Information 1 [file peerj-10-14009-s001.zip › Raw data/Figure 5D.png]

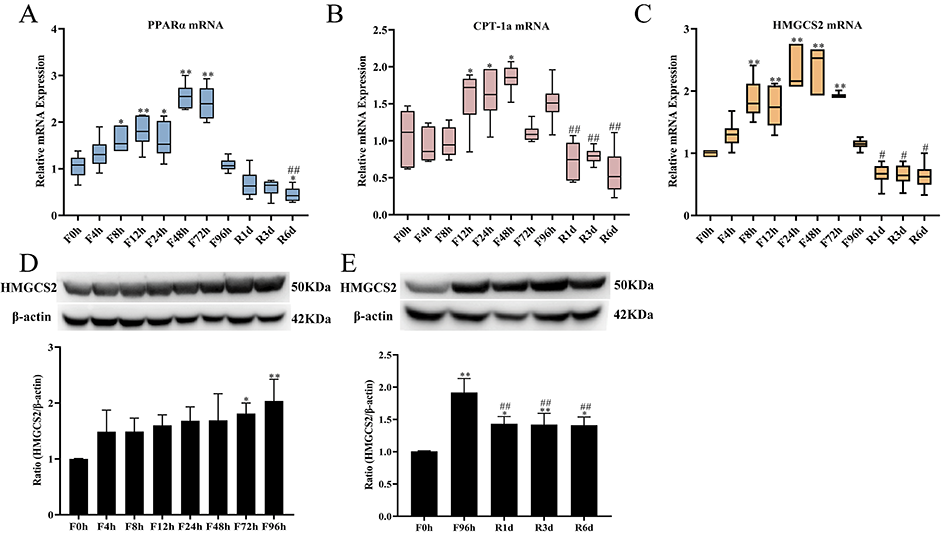

Supplement: Supplemental Information 1 [file peerj-10-14009-s001.zip › Raw data/Figure 6.png]

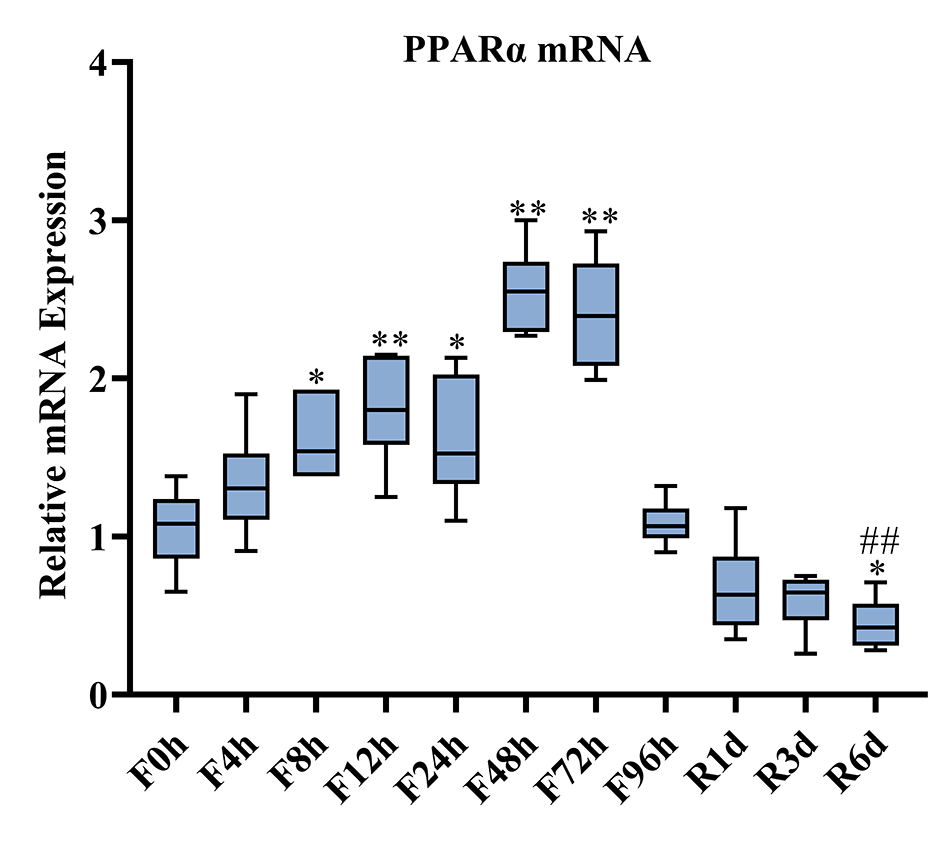

Supplement: Supplemental Information 1 [file peerj-10-14009-s001.zip › Raw data/Figure 6A.png]

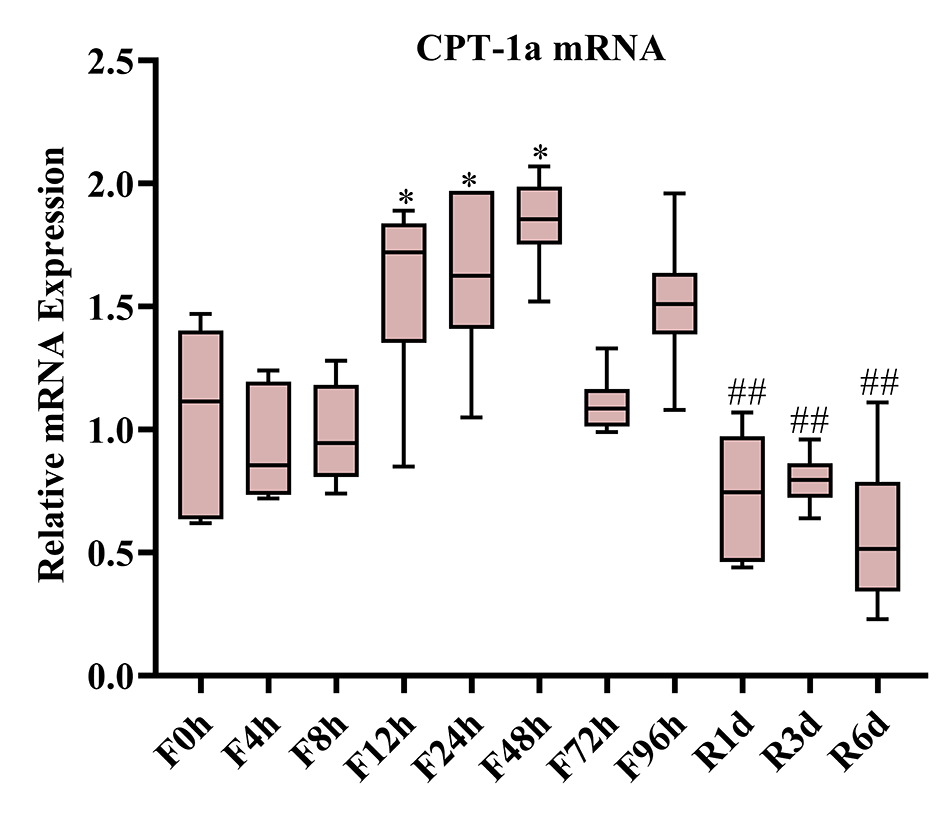

Supplement: Supplemental Information 1 [file peerj-10-14009-s001.zip › Raw data/Figure 6B.png]

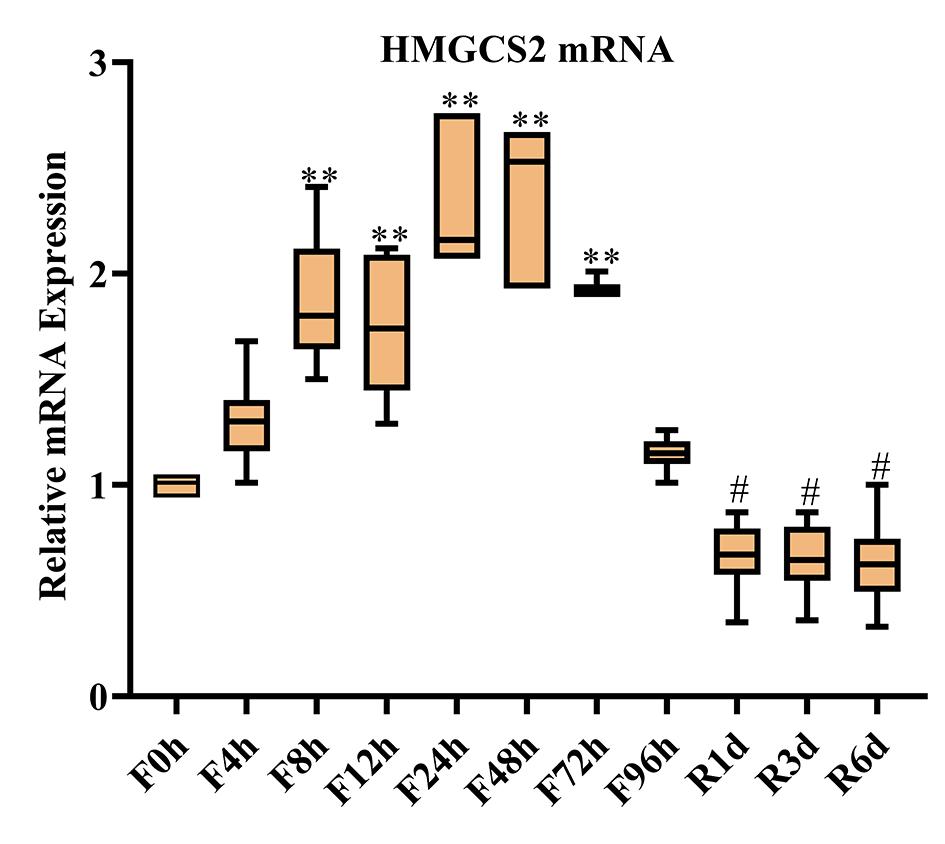

Supplement: Supplemental Information 1 [file peerj-10-14009-s001.zip › Raw data/Figure 6C.png]

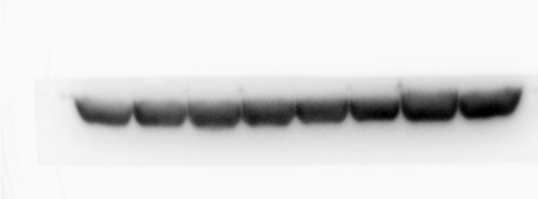

Supplement: Supplemental Information 1 [file peerj-10-14009-s001.zip › Raw data/Figure 6D HMGCS2-1.png]

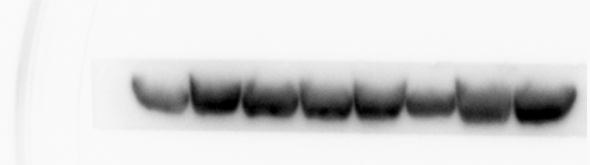

Supplement: Supplemental Information 1 [file peerj-10-14009-s001.zip › Raw data/Figure 6D HMGCS2-2.png]

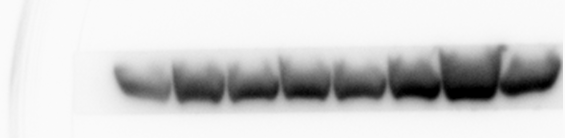

Supplement: Supplemental Information 1 [file peerj-10-14009-s001.zip › Raw data/Figure 6D HMGCS2-3.png]

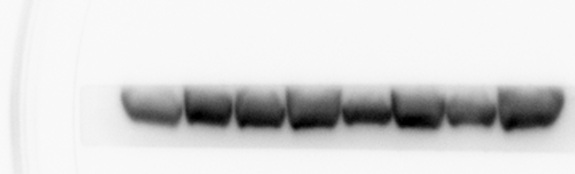

Supplement: Supplemental Information 1 [file peerj-10-14009-s001.zip › Raw data/Figure 6D HMGCS2-4.png]

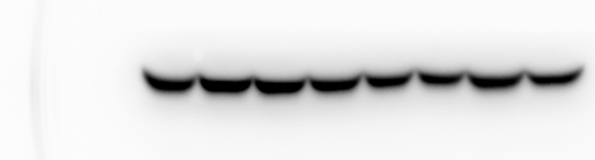

Supplement: Supplemental Information 1 [file peerj-10-14009-s001.zip › Raw data/Figure 6D a┬-actin-1.png]

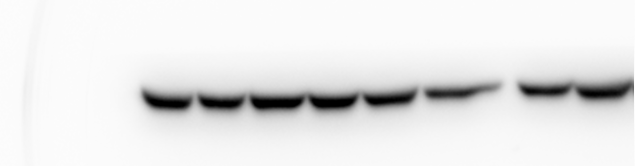

Supplement: Supplemental Information 1 [file peerj-10-14009-s001.zip › Raw data/Figure 6D a┬-actin-2.png]

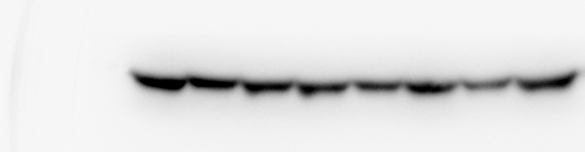

Supplement: Supplemental Information 1 [file peerj-10-14009-s001.zip › Raw data/Figure 6D a┬-actin-3.png]

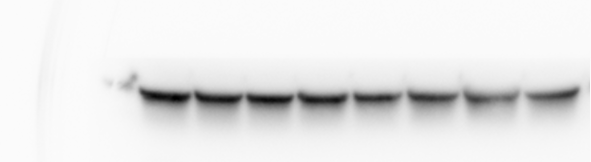

Supplement: Supplemental Information 1 [file peerj-10-14009-s001.zip › Raw data/Figure 6D a┬-actin-4.png]

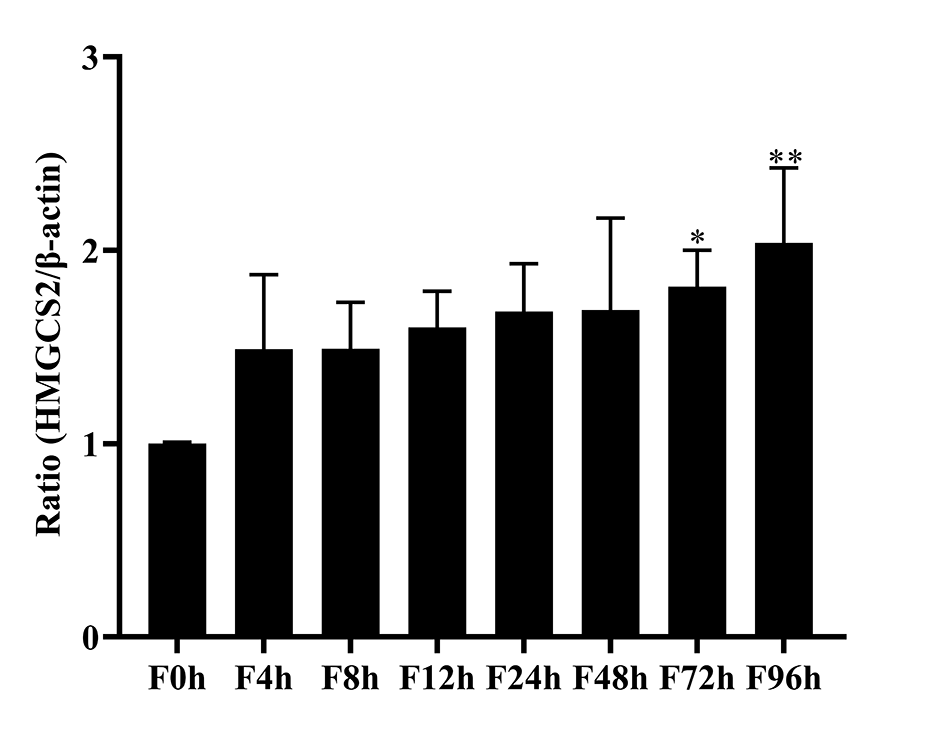

Supplement: Supplemental Information 1 [file peerj-10-14009-s001.zip › Raw data/Figure 6D.png]

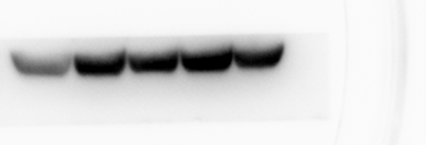

Supplement: Supplemental Information 1 [file peerj-10-14009-s001.zip › Raw data/Figure 6E HMGCS2-1.png]

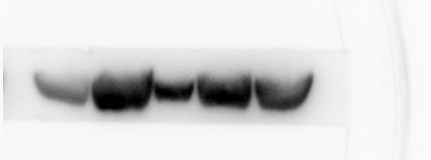

Supplement: Supplemental Information 1 [file peerj-10-14009-s001.zip › Raw data/Figure 6E HMGCS2-2.png]

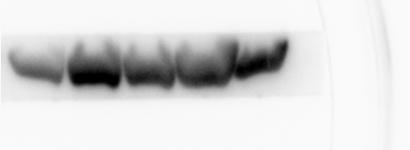

Supplement: Supplemental Information 1 [file peerj-10-14009-s001.zip › Raw data/Figure 6E HMGCS2-3.png]

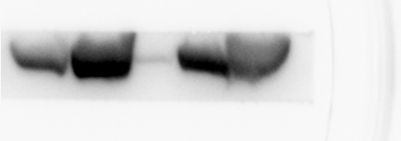

Supplement: Supplemental Information 1 [file peerj-10-14009-s001.zip › Raw data/Figure 6E HMGCS2-4.png]

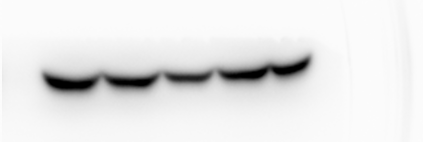

Supplement: Supplemental Information 1 [file peerj-10-14009-s001.zip › Raw data/Figure 6E a┬-actin-1.png]

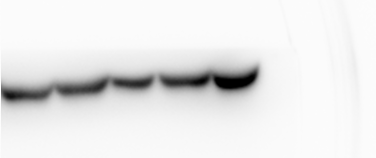

Supplement: Supplemental Information 1 [file peerj-10-14009-s001.zip › Raw data/Figure 6E a┬-actin-2.png]

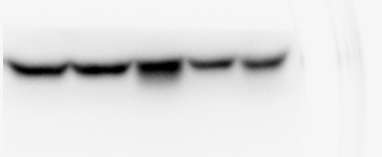

Supplement: Supplemental Information 1 [file peerj-10-14009-s001.zip › Raw data/Figure 6E a┬-actin-3.png]

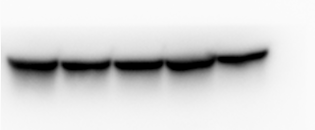

Supplement: Supplemental Information 1 [file peerj-10-14009-s001.zip › Raw data/Figure 6E a┬-actin-4.png]

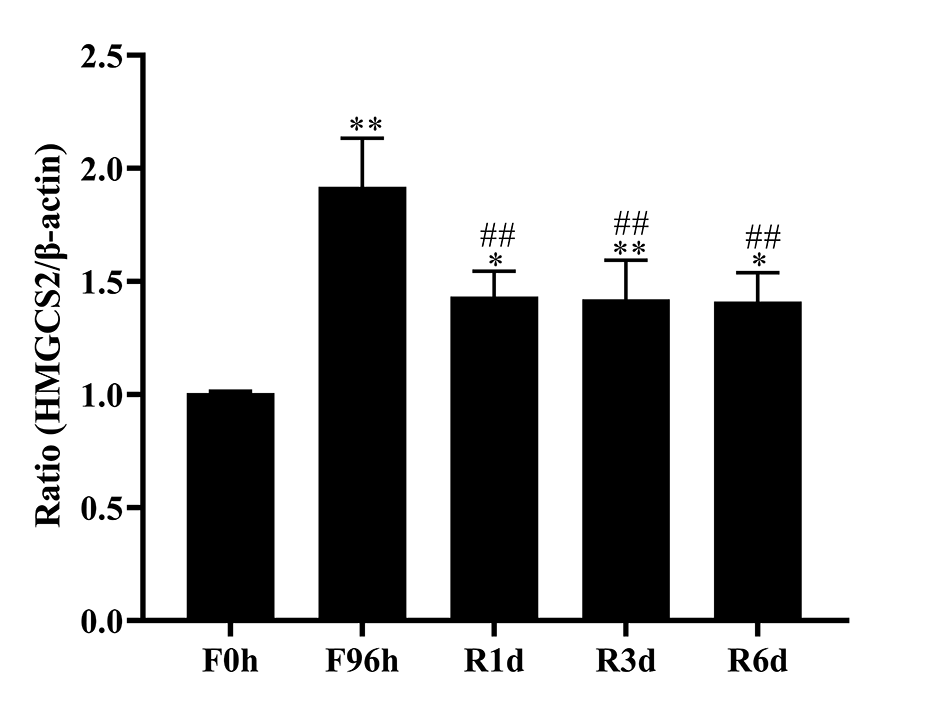

Supplement: Supplemental Information 1 [file peerj-10-14009-s001.zip › Raw data/Figure 6E.png]

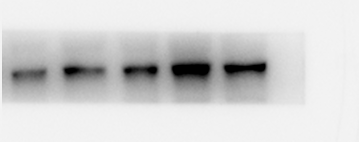

Supplement: Supplemental Information 1 [file peerj-10-14009-s001.zip › Raw data/Figure 7 I p-AMPK-1.png]

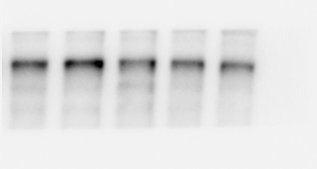

Supplement: Supplemental Information 1 [file peerj-10-14009-s001.zip › Raw data/Figure 7 I p-AMPK-2.png]

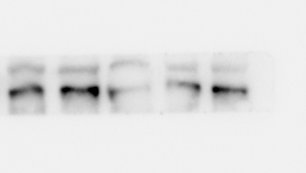

Supplement: Supplemental Information 1 [file peerj-10-14009-s001.zip › Raw data/Figure 7 I p-AMPK-3.png]

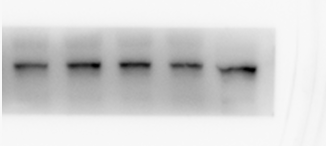

Supplement: Supplemental Information 1 [file peerj-10-14009-s001.zip › Raw data/Figure 7 I p-AMPK-4.png]

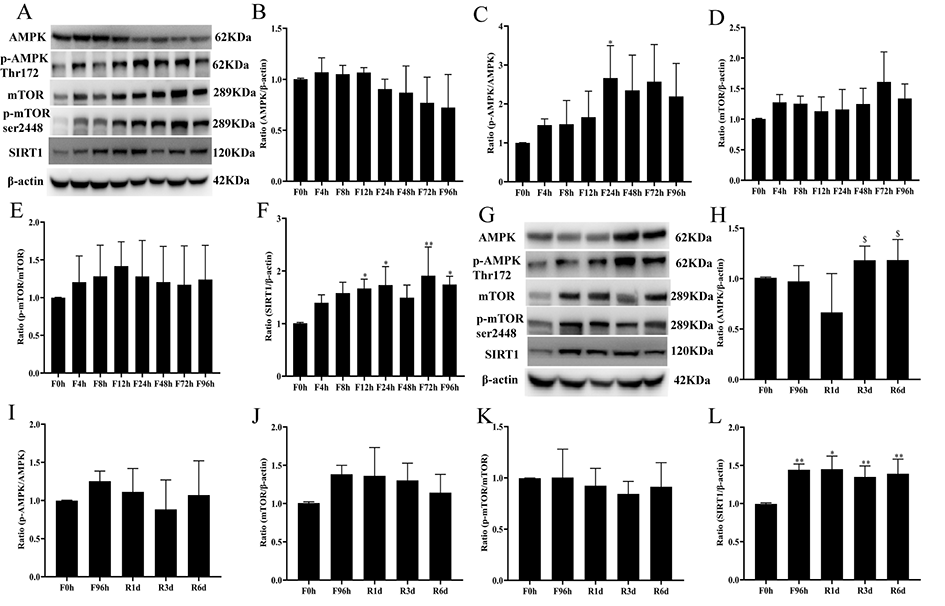

Supplement: Supplemental Information 1 [file peerj-10-14009-s001.zip › Raw data/Figure 7.png]

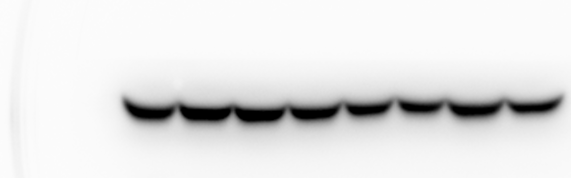

Supplement: Supplemental Information 1 [file peerj-10-14009-s001.zip › Raw data/Figure 7A a┬-actin-1.png]

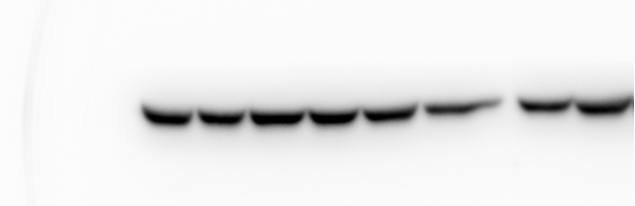

Supplement: Supplemental Information 1 [file peerj-10-14009-s001.zip › Raw data/Figure 7A a┬-actin-2.png]

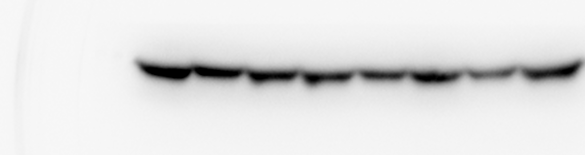

Supplement: Supplemental Information 1 [file peerj-10-14009-s001.zip › Raw data/Figure 7A a┬-actin-3.png]

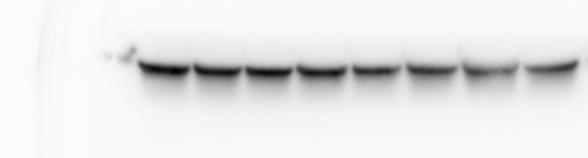

Supplement: Supplemental Information 1 [file peerj-10-14009-s001.zip › Raw data/Figure 7A a┬-actin-4.png]

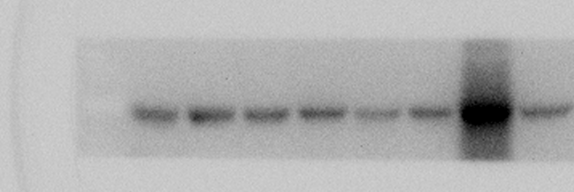

Supplement: Supplemental Information 1 [file peerj-10-14009-s001.zip › Raw data/Figure 7B AMPK-1.png]

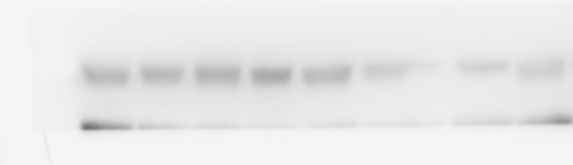

Supplement: Supplemental Information 1 [file peerj-10-14009-s001.zip › Raw data/Figure 7B AMPK-2.png]

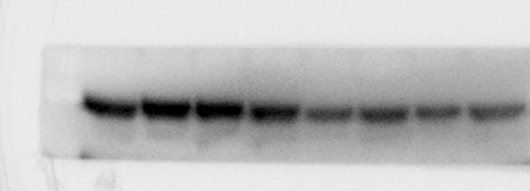

Supplement: Supplemental Information 1 [file peerj-10-14009-s001.zip › Raw data/Figure 7B AMPK-3.png]

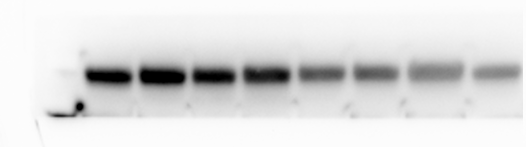

Supplement: Supplemental Information 1 [file peerj-10-14009-s001.zip › Raw data/Figure 7B AMPK-4.png]

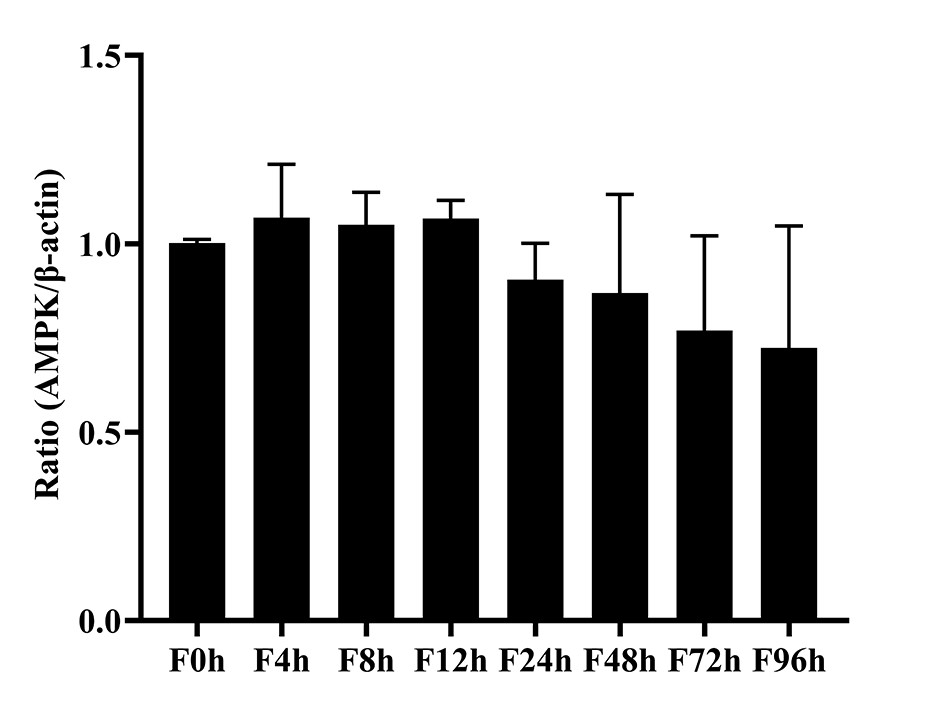

Supplement: Supplemental Information 1 [file peerj-10-14009-s001.zip › Raw data/Figure 7B.png]

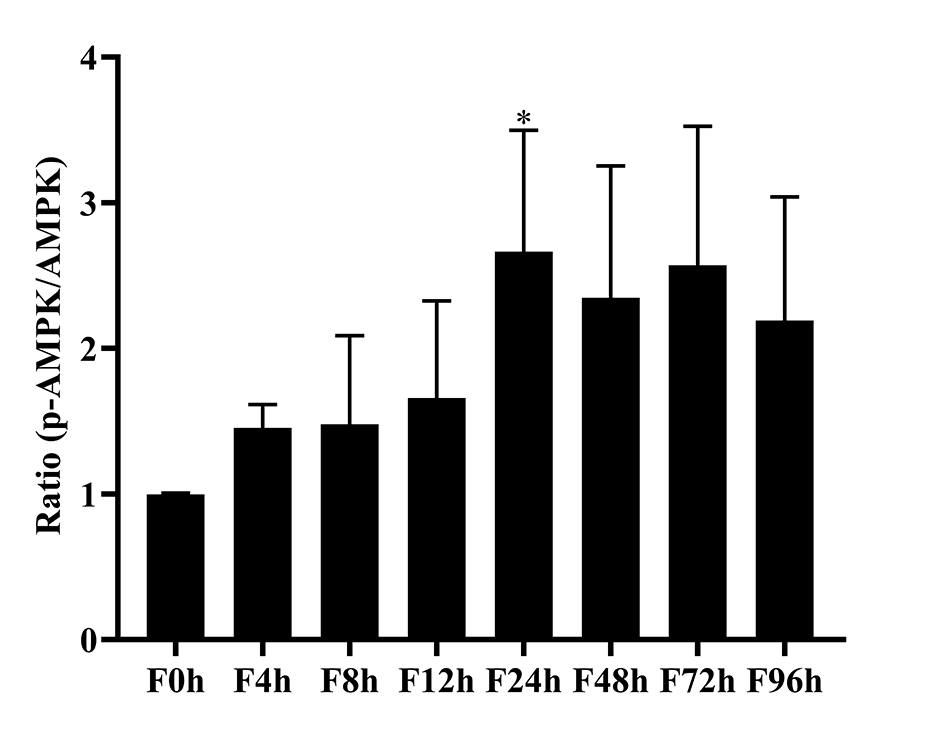

Supplement: Supplemental Information 1 [file peerj-10-14009-s001.zip › Raw data/Figure 7C.png]

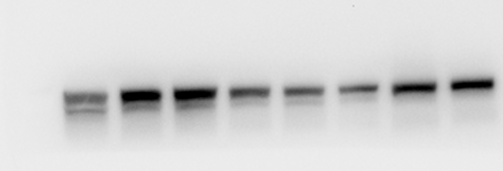

Supplement: Supplemental Information 1 [file peerj-10-14009-s001.zip › Raw data/Figure 7D mTOR-1.png]

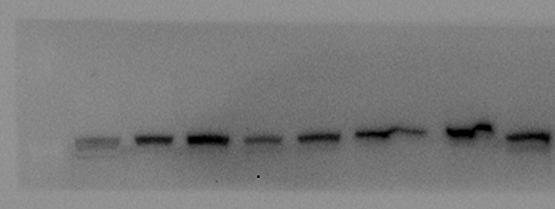

Supplement: Supplemental Information 1 [file peerj-10-14009-s001.zip › Raw data/Figure 7D mTOR-2.png]

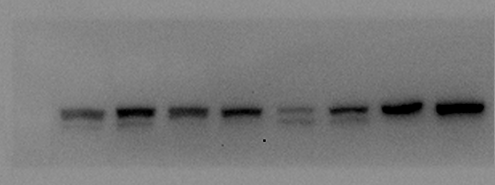

Supplement: Supplemental Information 1 [file peerj-10-14009-s001.zip › Raw data/Figure 7D mTOR-3.png]

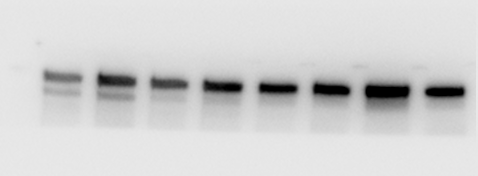

Supplement: Supplemental Information 1 [file peerj-10-14009-s001.zip › Raw data/Figure 7D mTOR-4.png]

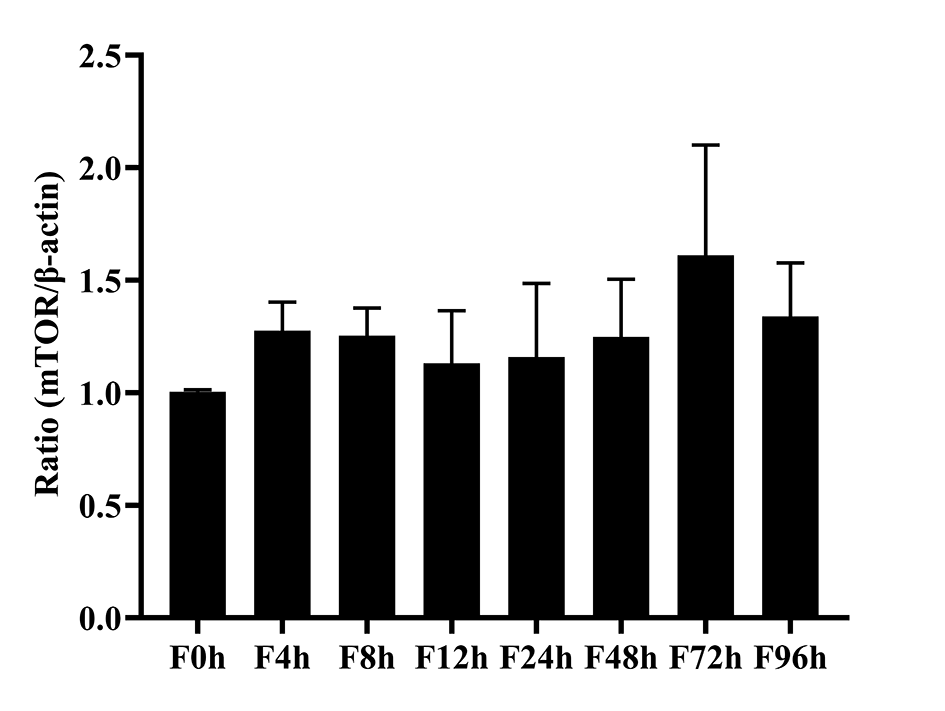

Supplement: Supplemental Information 1 [file peerj-10-14009-s001.zip › Raw data/Figure 7D.png]

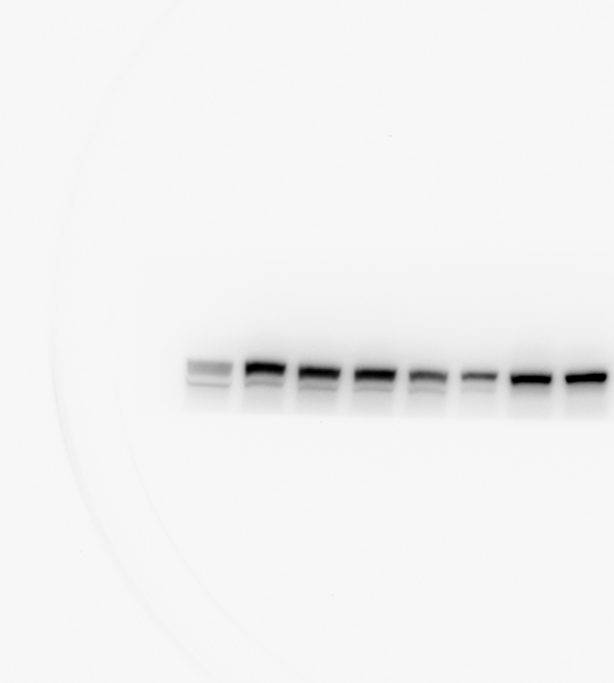

Supplement: Supplemental Information 1 [file peerj-10-14009-s001.zip › Raw data/Figure 7E p-mTOR-1.png]

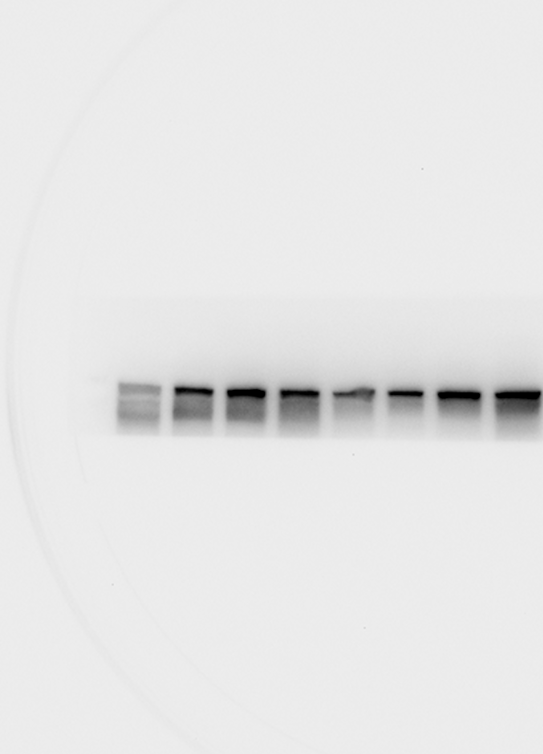

Supplement: Supplemental Information 1 [file peerj-10-14009-s001.zip › Raw data/Figure 7E p-mTOR-2.png]

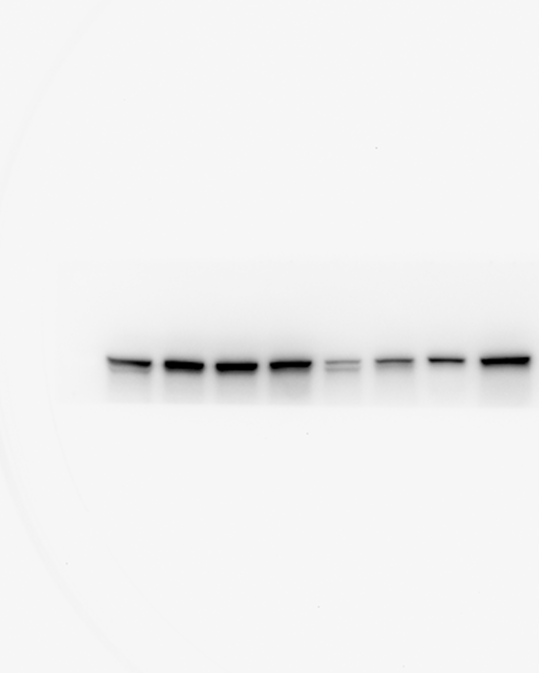

Supplement: Supplemental Information 1 [file peerj-10-14009-s001.zip › Raw data/Figure 7E p-mTOR-3.png]

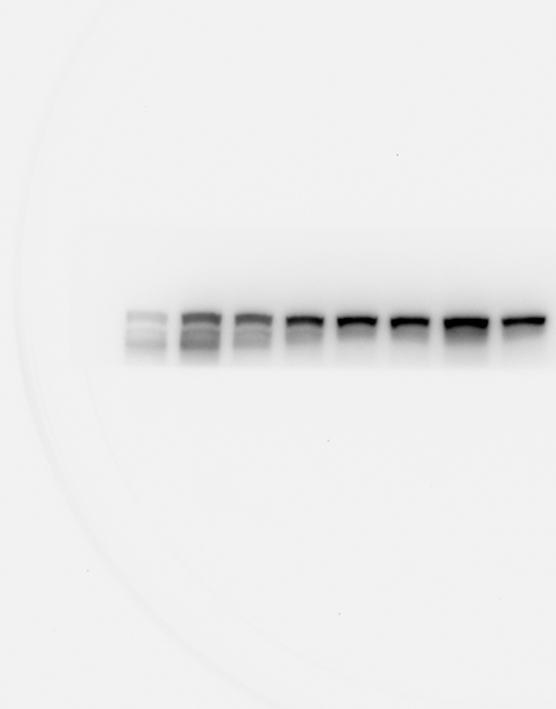

Supplement: Supplemental Information 1 [file peerj-10-14009-s001.zip › Raw data/Figure 7E p-mTOR-4.png]

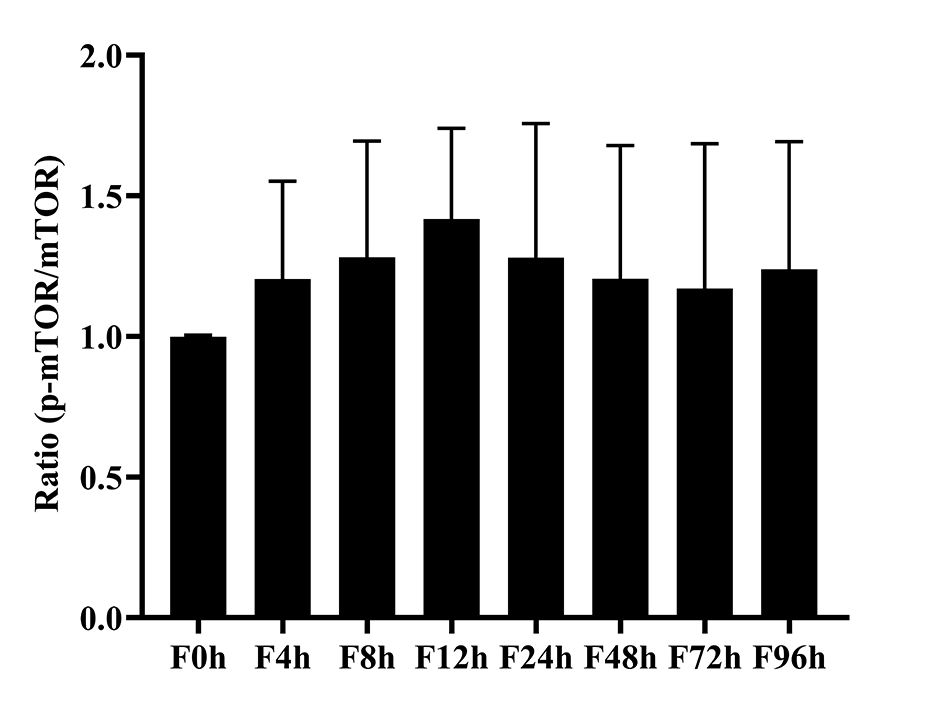

Supplement: Supplemental Information 1 [file peerj-10-14009-s001.zip › Raw data/Figure 7E.png]

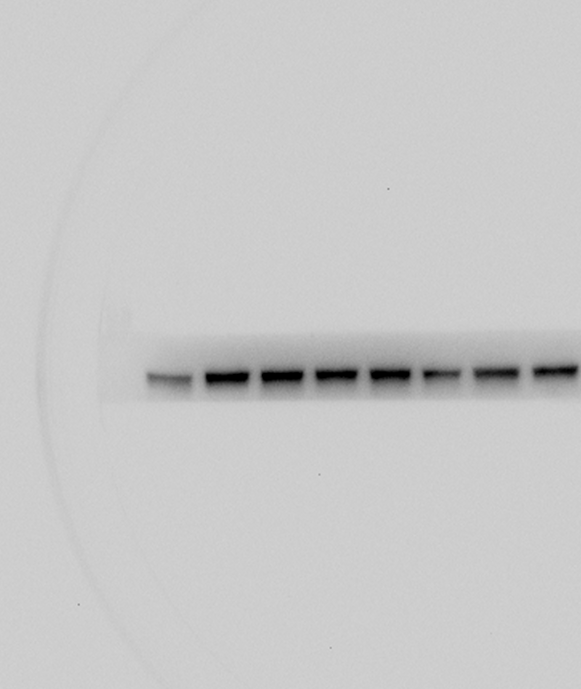

Supplement: Supplemental Information 1 [file peerj-10-14009-s001.zip › Raw data/Figure 7F SIRT1-1.png]

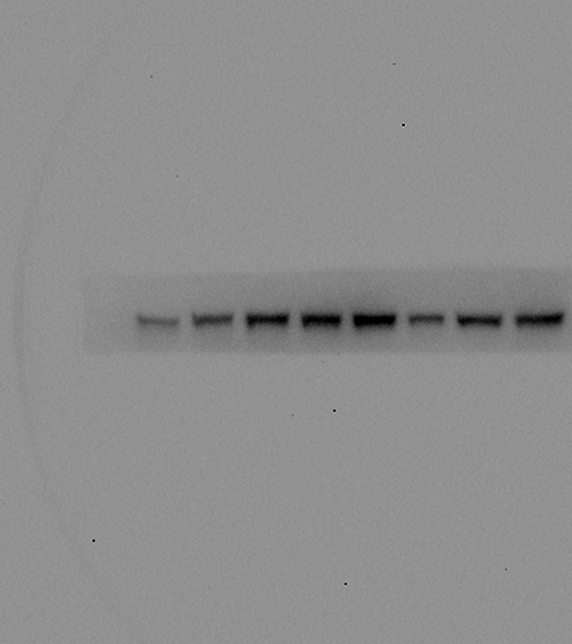

Supplement: Supplemental Information 1 [file peerj-10-14009-s001.zip › Raw data/Figure 7F SIRT1-2.png]

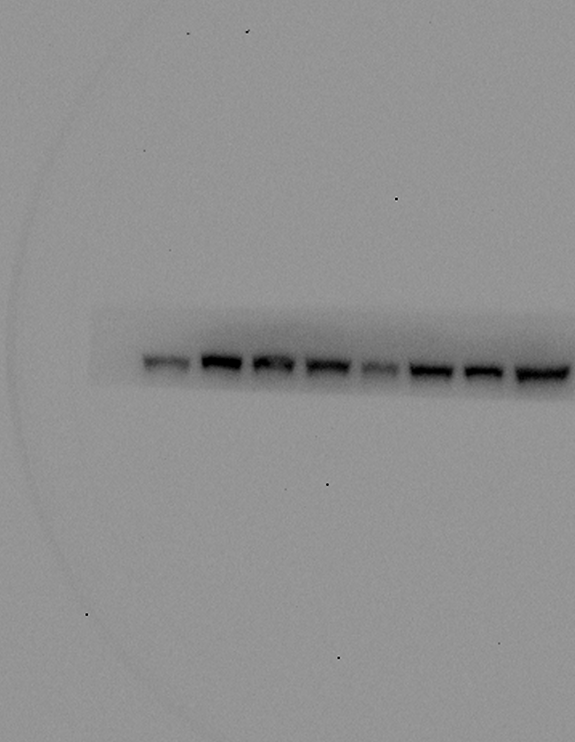

Supplement: Supplemental Information 1 [file peerj-10-14009-s001.zip › Raw data/Figure 7F SIRT1-3.png]

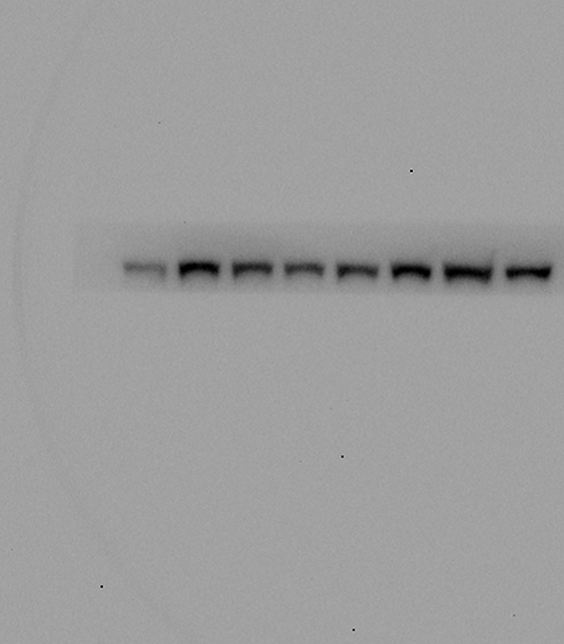

Supplement: Supplemental Information 1 [file peerj-10-14009-s001.zip › Raw data/Figure 7F SIRT1-4.png]

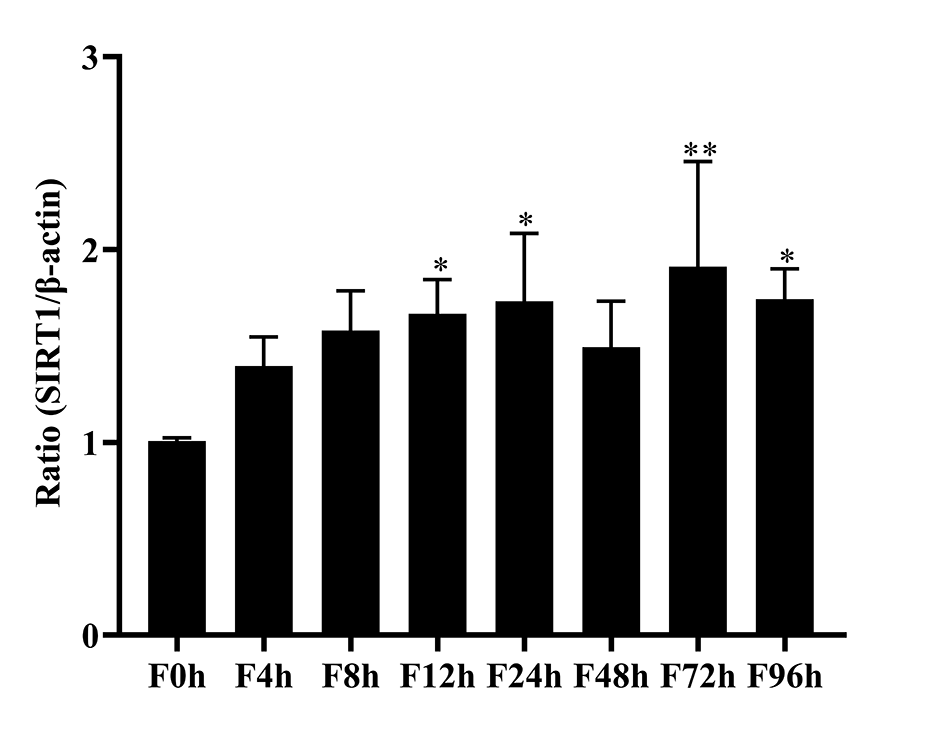

Supplement: Supplemental Information 1 [file peerj-10-14009-s001.zip › Raw data/Figure 7F.png]

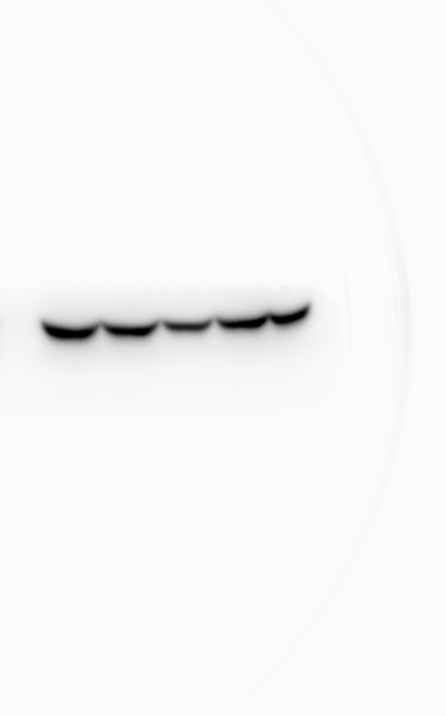

Supplement: Supplemental Information 1 [file peerj-10-14009-s001.zip › Raw data/Figure 7G a┬-actin-1.png]

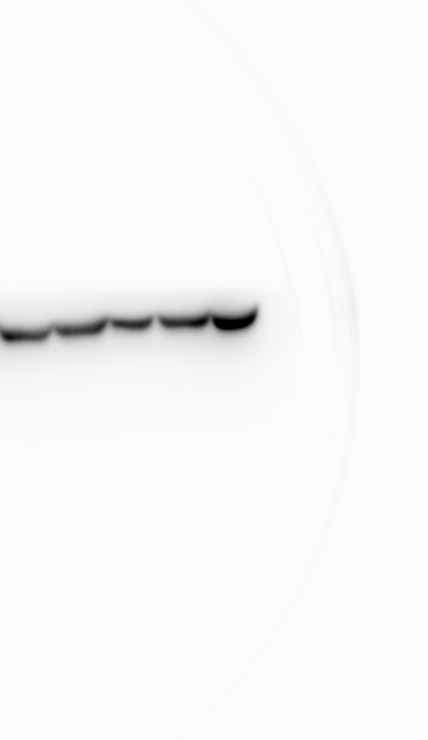

Supplement: Supplemental Information 1 [file peerj-10-14009-s001.zip › Raw data/Figure 7G a┬-actin-2-.png]

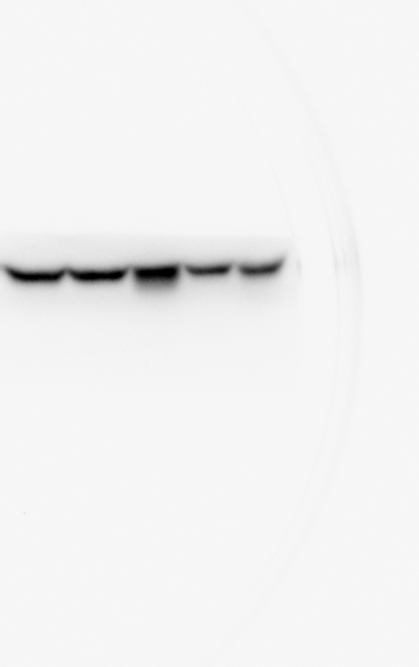

Supplement: Supplemental Information 1 [file peerj-10-14009-s001.zip › Raw data/Figure 7G a┬-actin-3.png]

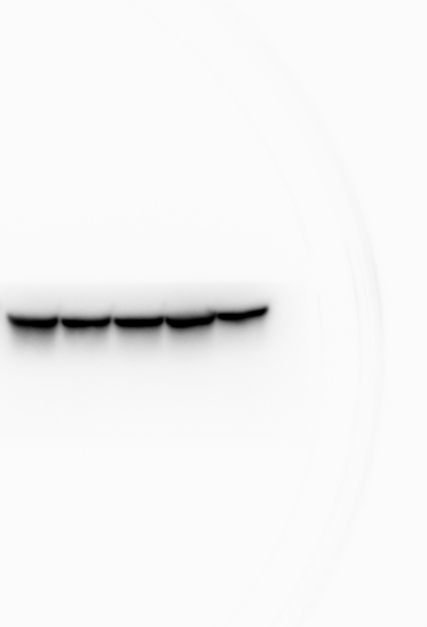

Supplement: Supplemental Information 1 [file peerj-10-14009-s001.zip › Raw data/Figure 7G a┬-actin-4.png]

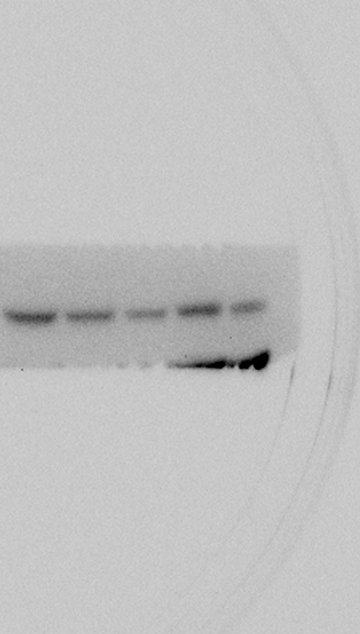

Supplement: Supplemental Information 1 [file peerj-10-14009-s001.zip › Raw data/Figure 7H AMPK-1.png]

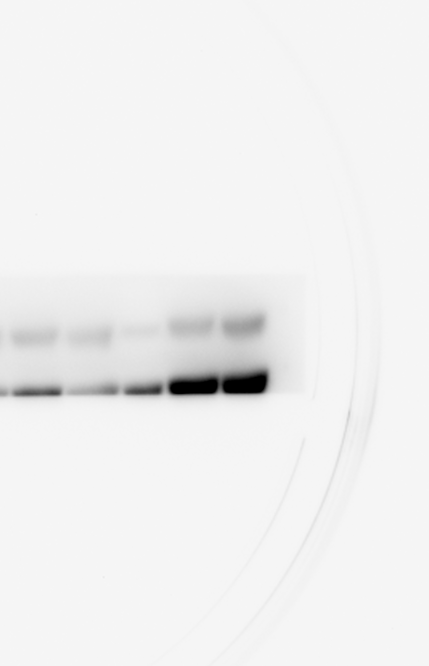

Supplement: Supplemental Information 1 [file peerj-10-14009-s001.zip › Raw data/Figure 7H AMPK-2.png]

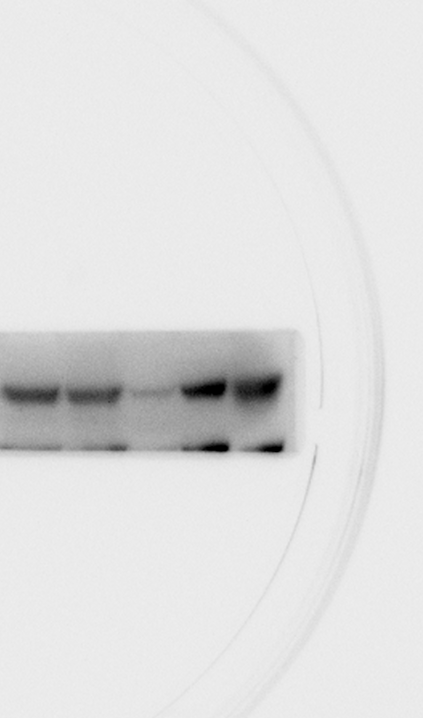

Supplement: Supplemental Information 1 [file peerj-10-14009-s001.zip › Raw data/Figure 7H AMPK-3.png]

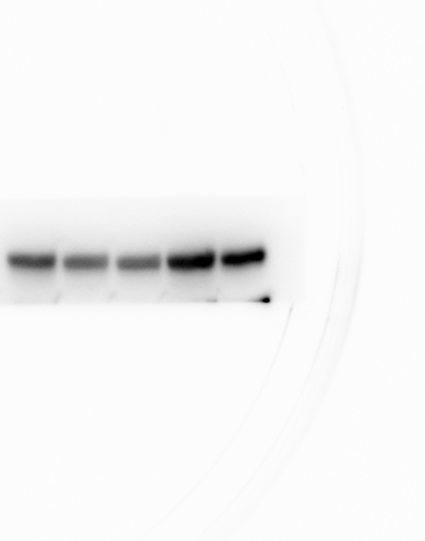

Supplement: Supplemental Information 1 [file peerj-10-14009-s001.zip › Raw data/Figure 7H AMPK-4.png]

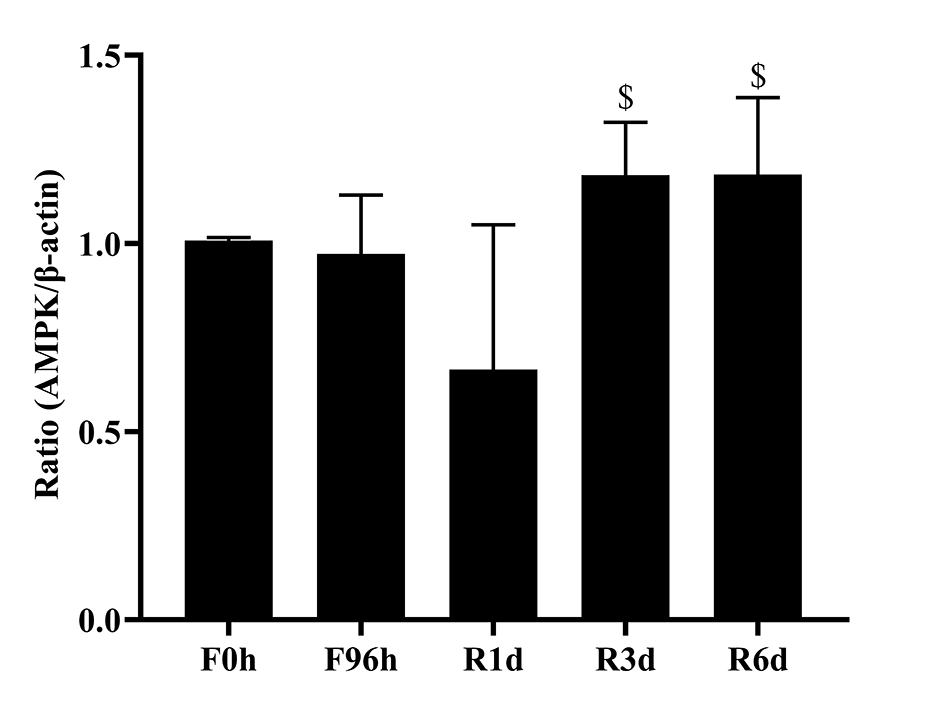

Supplement: Supplemental Information 1 [file peerj-10-14009-s001.zip › Raw data/Figure 7H.png]
